# Transcriptome and microRNA Sequencing Identified miRNAs and Target Genes in Different Developmental Stages of the Vascular Cambium in *Cryptomeria fortunei* Hooibrenk

**DOI:** 10.3389/fpls.2021.751771

**Published:** 2021-11-18

**Authors:** Hailiang Hu, Zhenhao Guo, Junjie Yang, Jiebing Cui, Yingting Zhang, Jin Xu

**Affiliations:** ^1^Key Laboratory of Forest Genetics and Biotechnology of Ministry of Education, Nanjing Forestry University, Nanjing, China; ^2^Co-Innovation Center for Sustainable Forestry in Southern China, Nanjing Forestry University, Nanjing, China; ^3^College of Forestry, Nanjing Forestry University, Nanjing, China; ^4^Jiangsu Key Laboratory for Horticultural Crop Genetic Improvement, Institute of Horticulture, Jiangsu Academy of Agricultural Sciences, Nanjing, China

**Keywords:** *Cryptomeria fortunei*, transcriptome, miRNA, vascular cambium, regulatory genes

## Abstract

*Cryptomeria fortunei* Hooibrenk is an important fast-growing coniferous timber species that is widely used in landscaping. Recently, research on timber quality has gained substantial attention in the field of tree breeding. Wood is the secondary xylem formed by the continuous inward division and differentiation of the vascular cambium; therefore, the development of the vascular cambium is particularly important for wood quality. In this study, we analyzed the transcriptomes of the cambial zone in *C. fortunei* during different developmental stages using Illumina HiSeq sequencing, focusing on general transcriptome and microRNA (miRNA) data. We performed functional annotation of the differentially expressed genes (DEGs) in the different stages identified by transcriptome sequencing and generated 15 miRNA libraries yielding 4.73 Gb of clean reads. The most common length of the filtered miRNAs was 21nt, accounting for 33.1% of the total filtered reads. We annotated a total of 32 known miRNA families. Some miRNAs played roles in hormone signal transduction (miR159, miR160, and miR166), growth and development (miR166 and miR396), and the coercion response (miR394 and miR395), and degradome sequencing showed potential cleavage sites between miRNAs and target genes. Differential expression of miRNAs and target genes and functional validation of the obtained transcriptome and miRNA data provide a theoretical basis for further elucidating the molecular mechanisms of cellular growth and differentiation, as well as wood formation in the vascular cambium, which will help improve the wood quality of *C. fortunei*.

## Introduction

*Cryptomeria fortunei* Hooibrenk belongs to the genus *Cryptomeria* in the *Taxodiaceae* family and is endemic to China. Its timber has excellent properties and is suitable for construction, furniture manufacturing, and other industrial applications, making it one of the most important afforestation tree species in China’s high-altitude areas. The emergence of tall woody plants is directly associated with secondary growth of stems, which includes the development of vascular cambium and the differentiation of cambium into secondary tissue.

Cambium cells can differentiate into secondary xylem and secondary phloem, thereby thickening the stem of the plant. In recent years, it has been reported that cambium activity shows a clear seasonal pattern (Mishima et al., 2014; Ding et al., 2016). With the increase in temperature and daylength in the summer, cambium cells gradually become more active, their cell number increases, and the cell wall becomes thinner, that is, they enter the peak stage of cellular proliferative activity. In autumn, with decreases in temperature and daylength, the rate of cambium divisions slows down and the cambium enters the mature stage. In winter, cellular divisions and other activities stop, and the cambium enters the dormant stage.

In plants, miRNAs bind to target genes with a higher degree of complementarity than in animals, generally with fewer than four mismatches, and plant miRNAs can bind to AGO proteins and form miRNA-RISC complexes to exert cleavage functions (Mi et al., 2008; Li et al., 2011). Based on earlier studies, it was believed that plant miRNAs function by cleaving target genes. However, based on a study revealing regulation of *AP2* protein expression by Arabidopsis miR172 without changes in mRNA expression, it was later proposed that plant miRNAs can also silence their target genes through translational repression (Aukerman and Sakai, 2003). Numerous experiments have demonstrated that translation inhibition is a widespread regulatory mechanism used by plant miRNAs (Gandikota et al., 2007; Alonso-Peral et al., 2010). Interestingly, miRNAs not only negatively regulate target genes but also can positively regulate their target genes. It has been experimentally demonstrated that miRNAs can positively regulate target genes by means of translational and transcriptional activation (Zhang et al., 2014; Xiao et al., 2017).

Recently, small RNA (sRNA) sequencing technology and bioinformatics approaches have been widely used for miRNA identification in plants. For example, sequencing of miRNAs and target gene prediction was performed for *Pinus massoniana Lamb* (Ye et al., 2020). A total of 364 known miRNAs and 117 novel miRNAs were found in *Cinnamomum bodinieri* (Chen et al., 2020a). A total of 239 and 369 known and novel miRNAs were identified in *Colletotrichum gloeosporioides* in tea plant (*Camelliasinensis L*.), and a miRNA-mRNA regulatory network was constructed (Jeyaraj et al., 2019).

At present, a wide range of topics related to *C. fortunei* is being studied, from macroforest ecology to microphysiology and even molecular genetics. Regarding molecular genetics, Luo et al. (2017) constructed a molecular fingerprint of 89 *C. fortunei* clones using 11 polymorphic SSR primers. *C. fortunei* genes related to cold resistance and lignin synthesis were identified and functionally studied, further advancing the exploration of *C. fortunei* gene function (Guo et al., 2019). In recent years, a large number of studies on the miRNA-mediated regulation of vascular cambium development have been published. For example, it was found that miR319a changes the lignin content during poplar secondary growth by regulating *PtoTCP20* (Hou et al., 2020). Significantly increased expression levels of miR164 and miR319 were observed by miRNA sequencing during the vascular cambial transition from the dormant stage to the active stage in *Ginkgo biloba* (Cui et al., 2016). One study also found that miR156 and miR172 play important roles in the development of the vascular cambium in *Cunninghamia lanceolata* (Qiu et al., 2015). However, few studies on the function of noncoding RNAs in *C. fortunei* have been published. Here, we used transcriptome sequencing to identify the function of differentially expressed miRNAs and their target genes during successive growth stages of *C. fortunei* vascular cambium. Our results further elucidate the molecular mechanism underlying the cellular growth and differentiation of the vascular cambium and provide a theoretical basis for exploiting the interactions between miRNAs and target genes to improve *C. fortunei* wood quality.

## Materials and Methods

### Plant Materials and Extraction of Total RNA

As previously described (Yang et al., 2020a), we acquired samples from *C. fortunei* trees aged approximately 60years with no obvious presence of insect pests or disease from the arboretum of Nanjing Forestry University, Nanjing City, Jiangsu Province, China (32°08′20.2″N,118°81′85.9″E). With a slight modification to Soile’s method (Soile et al., 2018), the vascular cambium tissue under the bark of the trunk was scraped with a blade immediately frozen in liquid nitrogen and then stored at −80°C. Samples were taken and numbered on April 20 (Group A), May 24 (Group B), June15 (Group C), July10 (Group D), and September 15 (Group E). In each group, three biological replicates were collected as materials for transcriptome sequencing and miRNA sequencing.

Total RNA was isolated from the *C. fortunei* vascular cambium using the RNeasy Plant Mini Kit (Qiagen, Hilden, Germany). Total RNA integrity and concentration were assessed by an Agilent 2100 Bioanalyzer (Agilent Technologies, Santa Clara, CA, United States) and a Thermo Scientific NanoDrop 2000 (Thermo Fisher Scientific, Wilmington, DE, United States). High-quality RNA was used for subsequent sequencing.

### Microscopic Observation

Fresh samples (0.5cm^3^) were fixed in formalin-acetic-50% alcohol (FAA, v/v) solution and then stained in safranin O and fast green FCF solutions. After staining, the samples were sealed with resin, and 8μm sections were observed under a microscope.

### Library Construction and Sequencing

Transcriptome sequencing was conducted by OE Biotech Co., Ltd. (Shanghai, China). For cDNA library construction, total RNA was digested with DNase and then enriched with oligo (dt) beads. mRNA was digested into short fragments and used as a template to synthesize cDNA. The synthesized cDNA was purified, end-repaired, supplemented with poly (A), ligated with Illumina sequencing adapters, selected based on fragment size, and then amplified by PCR. The constructed cDNA libraries were quality-checked with an Agilent 2100 Bioanalyzer and sequenced using an Illumina HiSeq X 10 sequencer. To obtain clean reads, we used Trimmomatic version 0.36 (Bolger et al., 2014) for the quality preprocessing of raw reads: Adapters were removed, and low-quality reads and low-quality bases were removed from the 3′ and 5′ ends, according to the following parameters: LEADING: 3, TRAILING: 3, SLIDINGWINDOW: 4:15, and MINLEN: 50. Clean reads were spliced into *de novo* transcripts by using Trinity version 2.4.0 (Grabherr et al., 2011) in paired-end mode (parameters: SS_lib_type RF). Then, the longest transcript was selected as a unigene based on sequence similarity and sequence length.

To construct 15 sRNA libraries, adaptor sequences, N-base sequences, sequences over 15–41nt in length, and low-quality sequences were removed from the raw reads to obtain high-quality clean reads. The clean reads included miRNAs, transfer RNAs, ribosomal RNAs, and small nuclear RNAs. Length statistics were performed on small RNAs to determine the length distribution. To classify and annotate small RNAs, clean reads were aligned and annotated with Rfam version 10.0^1^ and miRbase version 22.0 (Griffiths, 2008).^2^

To construct a degradation library, mRNAs were captured by magnetic beads ligated with 3′ and 5′ adaptors and reverse-transcribed by using a mixture of biotinylated random primers and mRNAs. Finally, the constructed library was sequenced in an Illumina HiSeq 2,500 sequencer (Illumina, San Diego, CA, United States), and the single end read length was 1×50bp.

### miRNA Sequence Alignment and Annotation

After removing the tRNA, rRNA, snRNA, and other sRNA sequences from the sRNA sequencing data by using Bowtie (Langmead, 2010), the filtered sequences were compared to the miRBase version 22.0 database to identify known miRNAs. miRDeep2 version 2.0.0.8 (Friedländer et al., 2012) was used to predict novel miRNAs. The secondary structure of the identified miRNAs was predicted using RNAfold^3^ (Denman, 1993), and sequences capable of forming a miRNA hairpin precursor were flagged as potential miRNA sequences. The mature and star (complementary strand) sequences of the predicted miRNAs were extracted, and the novel miRNAs were analyzed quantitatively.

### Differential Expression Analysis, miRNA Target Gene Prediction and Functional Annotation

miRNA expression was quantified using the known and predicted miRNAs as a reference. The transcript per million (TPM) index was used to calculate miRNA expression (Sun et al., 2014), and differentially expressed genes (DEGs) were identified using DESeq version 1.26.0 with the following parameters: *p*<0.05 and |log2FoldChange|>1 (Storey and Tibshirani, 2003; Love et al., 2014). To obtain clean tags and cluster tags, the miRNA sequencing results were filtered for low-quality tags, and connector sequences were removed. Annotating information for noncoding RNA was obtained by comparing cluster tags to the Rfam version 10.0 (see footnote 1) database using TargetFinder to predict plant target genes (Fahlgren and Carrington, 2010; Ding et al., 2012) with default parameters. To further study the functions of miRNAs and target genes, it is necessary to analyze miRNAs and target genes by using KEGG and GO analyses. Then, Cytoscape version 3.5.1 was used to construct an interaction network between miRNAs and target genes.

### Gene Expression Validation Using qRT-PCR

Primers specific for the chosen gene sequences were designed using Primer Premier (version 5.00) software (Supplementary Table 1). Three commonly used internal reference genes (*UBI*, *GAPDH*, and *18S rRNA*) were selected as candidate internal reference genes, and the Ct values of candidate internal reference genes were obtained and analyzed by geNorm, NormFinder, and BestKeeper (Vandesompele et al., 2002; Andersen et al., 2004; Pfaffl et al., 2004) to select the best internal reference genes. Vazyme miRNA Design (v1.01) software was used to design primers for the real-time fluorescence quantification of miRNAs, and primers for first-strand cDNA synthesis were synthesized using the stem-loop method. The designed primers are shown in Supplementary Table 1.

The *C. fortunei* reverse transcription cDNA products and the first-strand cDNA products were diluted 10 times, and 3 replicates were analyzed for each sample and internal reference. Then, according to the kit’s protocol, qPCR was carried out on an ABI 7500 Step OnePlus Real-time PCR System (Applied Biosystems, Foster City, CA, United States), with the following conditions: 95°C for 20s, 95°C for 10s, 95°C for 10s, and 60°C for 34s.

## Results

### Cell Morphological Changes in the Vascular Cambia at Different Growth Stages

**Figure 1:**
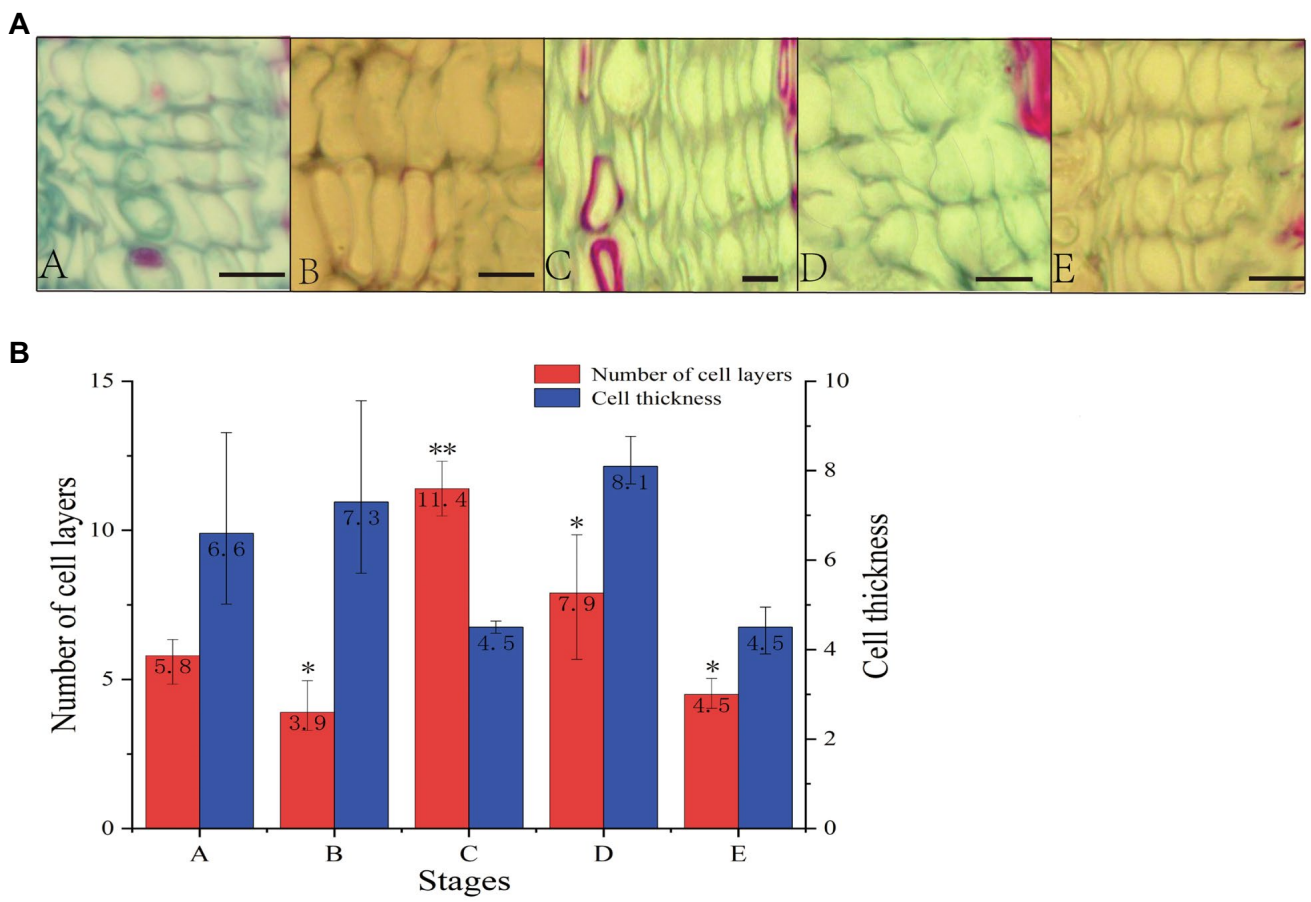
**(A)** Cross section of stem segments of *C. fortunei* (A: April 20th, B: May 24th, C: June 15th, D: July 10th, E: September 15th; bar=10μm). **(B)** Number of cell layers and cell thickness statistical analysis. The x-axis represents the different stages, and the y-axes on the left and right represent the number of cell layers and cell thickness, respectively; ^*^p <0.05, ^**^p <0.01.

To study vascular cambium growth and development in *C. fortunei*, we observed and analyzed cambium cells at different growth stages (Figure 1). In stages A–E, the average number of vascular cambium cells first increased and then decreased and reached its highest value in the C stage. The average number of vascular cambium cells was 11.4 layers, and it was speculated that the growth and development of vascular cambium cells were strong in this stage. The cell wall of stage B cells was thinner, the cells were fuller, and the average number of cell layers was the lowest, and this may have prepared the tissue for the active growth of stage C cells. In the D and E stages, cell division gradually decreased, cell thickness decreased, and cell growth tended to flatten, and it was speculated that cambium cells gradually entered the maturation phase.

### Go and KEGG Enrichment Analyses of the mRNAs

**Figure 2:**
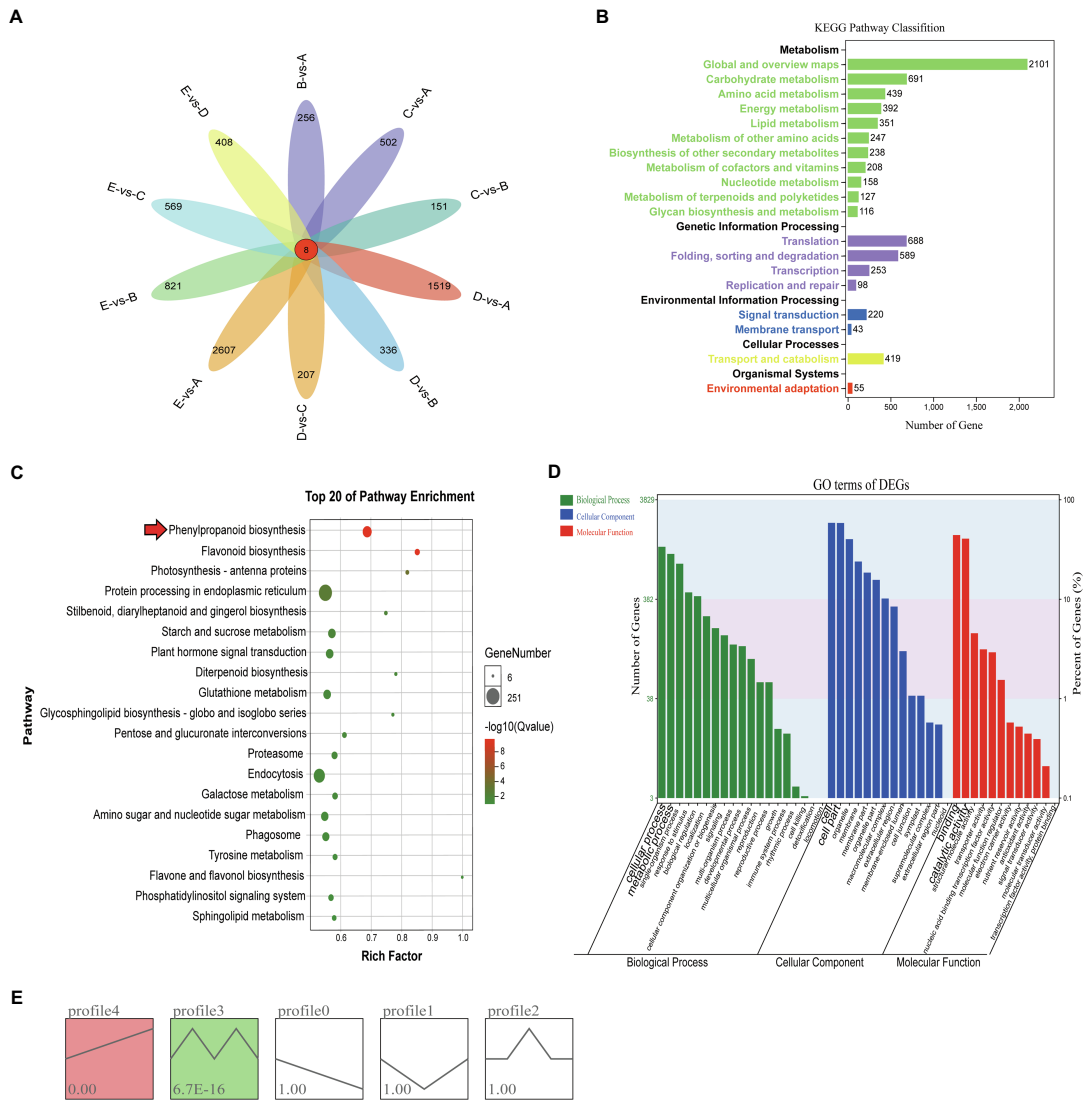
**(A)** Venn diagram of differentially expressed genes. **(B)** KEGG analysis of DEGs. The x-axis shows the number of DEGs annotated to each pathway; the y-axis indicates the name of the pathway, and the number on the right side of the column indicates the number of DEGs annotated to each pathway. **(C)** Top 20 enrichment pathways of DEGs. The x- and y-axes represent the enrichment factor and the pathway term, respectively. The colors and sizes of the dots represent the significance and the number of DEGs. **(D)** GO terms of DEGs. The x-axis presents the different GO functional classifications, and the y-axes on the left and right represent the number and percentage of DEGs classified in each GO category, respectively. **(E)** Different expression patterns of all genes. Each box represents a model profile. The upper number in the box represents the serial number of profiles, and the lower number is the value of *p*. Boxes with *p*<0.05 are colored.

A large number of DEGs were identified in different growth stages of vascular cambium development by transcriptome sequencing, and pairwise comparison of each stage revealed that each had eight identical DEGs, with EvsA having the highest number of DEGs (2615) and CvsB having the least number of DEGs (159; Figure 2A). To further study their function, the DEGs from all periods were combined for KEGG pathway annotation, and a large number of DEGs were found to be involved in global and overview maps, carbohydrate metabolism, translation and folding, sorting, and degradation (Figure 2B). Among the top 20 pathways enriched by KEGG, protein processing in the endoplasmic reticulum, endocytosis, plant hormone signal transduction, and phenylpropanoid biosynthesis were the most abundant (Figure 2C). Phenylpropanoid biosynthesis and flavonoid biosynthesis were highly significant (Supplementary Figure 1). This reveals that these pathways may be involved in the regulation of vascular cambium development. We also performed GO annotation and evaluated the DEGs according to the three categories of biological process, cellular component, and molecular function. We found that the DEGs were mainly involved in the cellular process and metabolic process of the biological process category, in the cell and cell binding terms of the cellular component category, and in the cell and catalytic activity of the molecular function category (Figure 2D). To compare the expression trends of these DEGs, we performed STEM cluster analysis, which yielded five profiles. Profiles 3 and 4 were significant, and profile 4 had an increasing trend, profile 0 had a decreasing trend, and profiles 1, 2, and 3 showed a change in trend at different stages (Figure 2E).

### Coexpression Network Analysis of the DEGs

**Figure 3:**
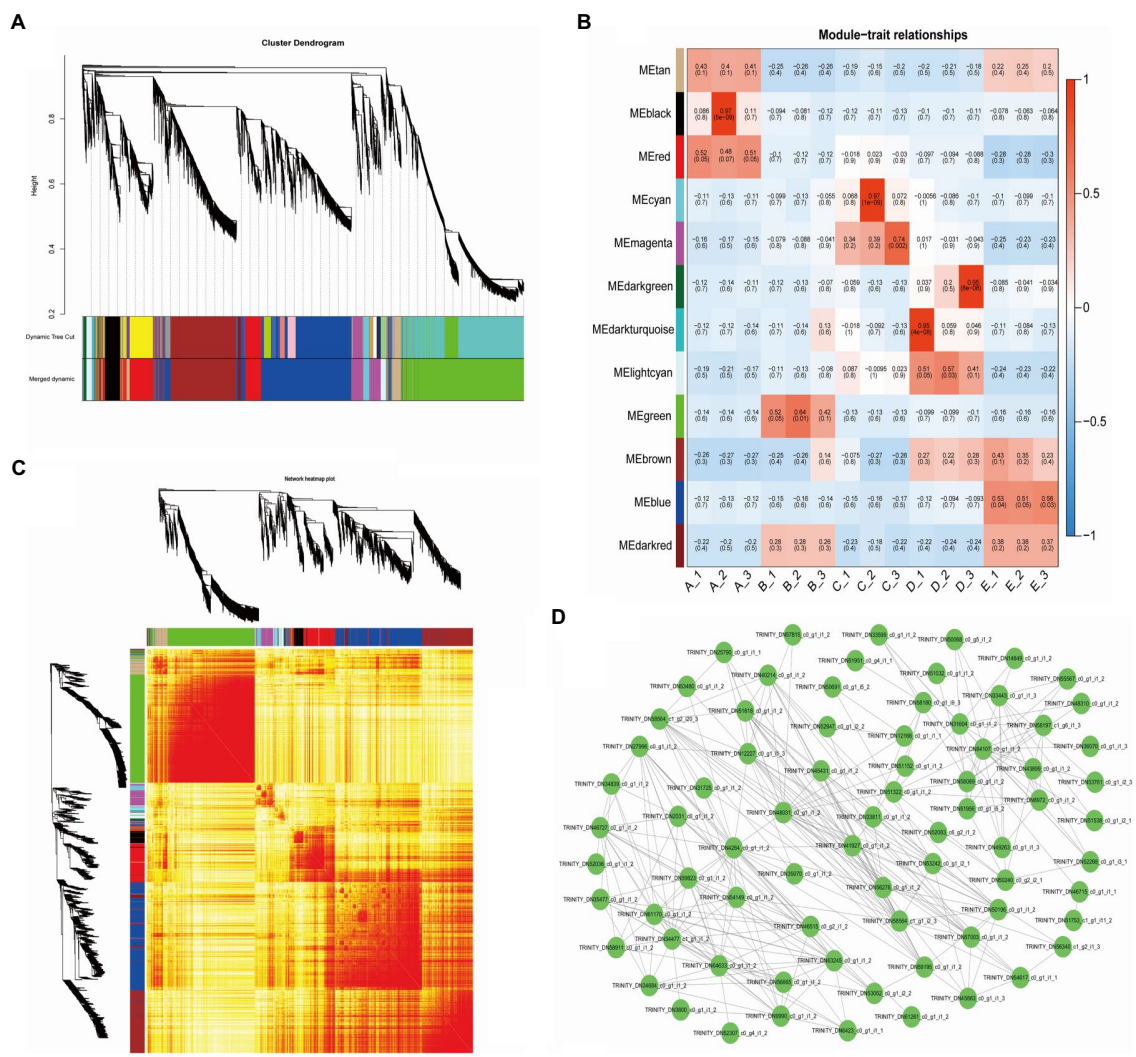
Weighted gene coexpression network analysis of DEGs. **(A)** Cluster dendrogram of 12 different expression modules. **(B)** Module-trait relationships. The x-axis shows the sample, and the y-axis shows the modules and significance, respectively. **(C)** Network heatmap plot. **(D)** Network of MEmagenta module DEGs.

To understand the correlation and expression trends of the DEGs in all samples, we used the FPKM values of a total of 29,617 DEGs in 15 samples (3 replicates in 5 stages) to construct the coexpression network, resulting in a total of 12 modules. Different colors represent different modules and contain three highly correlated gene clusters (Figure 3A). Seven of the modules showed specificity in temporal gene expression, the “black” and “red” modules were significantly expressed mainly in stage A, the “green” module was significantly expressed in stage B, the “cyan” and “magenta” modules had significant expression in stage C, and “dark green” and “blue” modules showed expression specificity in stages D and E (Figure 3B). We plotted the cluster heatmap for all genes and found that the genes within each module had a high correlation (Figure 3C). We used the “magenta” module of the C stage with vigorous vascular cambium development as an object to map the network of the 75 DEGs using Cytoscape software (Figure 3D) and found that these genes were mainly associated with protein processing in the endoplasmic reticulum, biosynthesis of secondary metabolites, and plant hormone signal transduction by functional annotation. The biosynthesis of secondary metabolites and plant hormone signal transduction play an important role in the development of vascular cambium and the formation of secondary tissues.

### sRNA Sequencing of Different Cambial Developmental Stages of *C. fortunei*

To understand the expression dynamics of *C. fortunei* miRNAs during cambial zone development, we constructed miRNA sequencing libraries using samples from five stages. Sequencing fragments were evaluated and filtered for base quality and length, and then, filtered read statistics are shown in Table 1. The Q30 values of all groups were greater than 90%, indicating the reliability of the data.

**Table 1:** Statistical analysis of clean reads.

Sample	Raw reads (M)	Number of reads 15\41nt in length (M)	Q30 (%)^*^	Number of reads without N-base (M)	Clean reads (M)	Clean reads unique (M)
A_1	24.08	11.35	93.64	11.14	11.14	2.42
A_2	34.97	13.61	93.96	13.34	13.34	2.76
A_3	31.39	9.73	93.55	9.53	9.53	1.97
B_1	22.85	15.16	93.11	14.90	14.90	2.79
B_2	22.84	13.87	92.91	13.61	13.61	2.73
B_3	24.55	15.37	92.33	15.12	15.12	2.84
C_1	23.38	15.54	94.45	15.30	15.30	3.51
C_2	26.89	12.46	94.33	12.21	12.21	2.68
C_3	28.13	14.13	94.30	13.88	13.88	3.23
D_1	25.33	14.96	93.01	14.69	14.69	3.35
D_2	26.41	15.61	94.85	15.32	15.32	3.56
D_3	21.61	13.53	92.99	13.28	13.28	3.26
E_1	17.54	14.51	93.20	14.26	14.26	2.54
E_2	18.75	15.28	92.51	14.89	14.89	2.53
E_3	19.28	16.28	94.89	15.99	15.99	3.12

* Q30 represents the percentage of bases whose phred number is greater than 30 among the raw bases.

**Figure 4:**
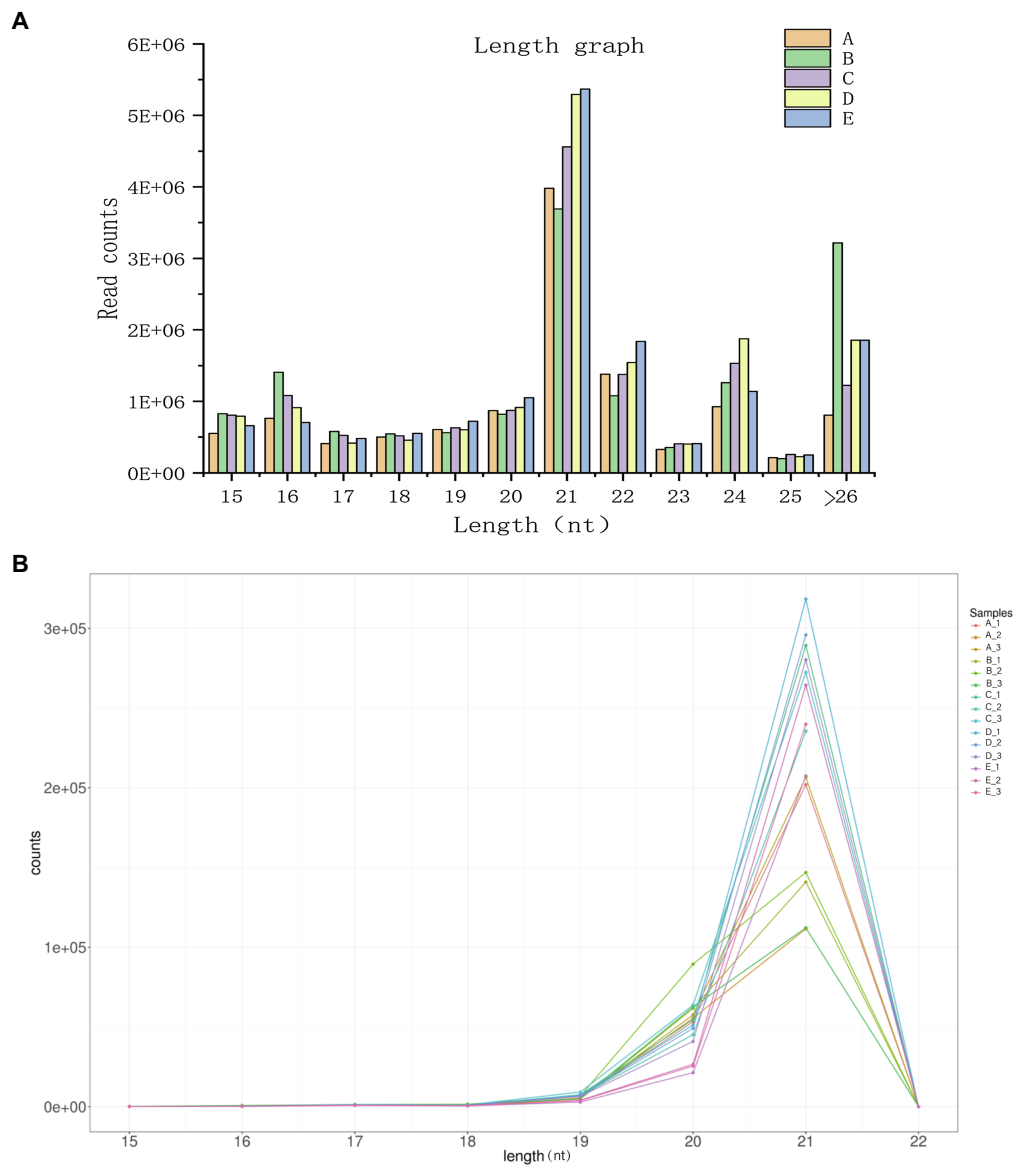
**(A)** Length distribution statistics of clean reads. The x-axis represents the size of the small RNA, and the y-axis represents the number of read counts. **(B)** Known miRNA length distribution line graph. The x-axis presents the size of the miRNA, and the y-axis presents the number of read counts.

To facilitate the processing of different samples after read filtering, we determined the sRNA length distribution, which will help us to better study the function of vascular cambium miRNAs (Figure 4A). The most frequent sRNA length in plants was 21nt, accounting for 33.1% of the total filtered reads. As shown in Table 2, rRNA, tRNA, snRNA, Cis-reg, and other Rfam annotations accounted for 0.02, 0.01, 0.03, 0.16, and 0.33% of the total reads, respectively, or 0.55% of all the reads in total. After removing these types of additional sRNA sequences, the known miRNAs accounted for 1.76% of the total reads, and unannotated sequences accounted for 93.72% of the total reads, which were used for the prediction and analysis of novel miRNAs.

**Table 2:** Sample read sequence types and attributes.

Annotation type	Sequence number	Percentage of total sequences (%)	Number of uniqsequences	Percentage of uniqsequences (%)
rRNA	3024	0.02	530	0.02
tRNA	1113	0.01	153	0.01
snRNA	3940	0.03	664	0.02
Cis-reg	23,708	0.16	678	0.02
other_Rfam_RNA	49,286	0.33	1035	0.04
gene	177,551	1.19	25,781	0.92
repeat	414,300	2.78	34,235	1.23
known_miRNA	263,040	1.76	243	0.01
Unannotated	13,968,271	93.72	2,726,380	97.73

### Identification of Known and Novel miRNAs

Plant miRNAs are highly evolutionarily conserved, and miRNA data from newly sequenced plants can be compared *via* alignment analysis to those of related species that have been previously recorded in the miRBase (version 22.0) database. For this study, we selected sequences from *Picea abies*, a species related to *C. fortunei*. The length distribution of known miRNAs is shown in Figure 4B, making it clear that among the known miRNAs, a length of 21nt is still the most abundant class and that the majority of known miRNAs are between 19–22 nt in length. After sequence alignment, we found a total of 80 conserved miRNAs belonging to 32 families, as shown in Supplementary Table 2. The most abundantly expressed families were miR396, miR159, and miR319, while the least abundant families were miR946, miR399, and miR529. Most miRNA families had multiple members that aligned to known sequences, with the miR399 family having the highest number of aligned miRNAs (10 in total). We used the TPM value (for each miRNA gene, this value indicates the number of miRNAs per million reads) as an indicator of miRNA expression. As shown in Supplementary Table 3, we found significant differences among the TPM values of different miRNAs at different cambium developmental stages. For example, miR396g was expressed in each of the stages, with its expression in stage B being the lowest (TPM=6904.47), while the TPM expression value of miR946c was less than 0.57 in each stage, which indicates that different miRNAs show a unique pattern of expression during vascular cambium development. Additionally, we found that transcription levels of individual members of the same miRNA family showed extensive variation during the 5 developmental stages we examined. For example, the expression level of miR396b varied between 321 and 2014 TPM, while that of miR396f remained very low (TPM values between 1 and 9), indicating that the expression abundance of known miRNA families in *C. fortunei* can vary to a large degree. Additionally, there were miRNAs with low levels of expression that showed no significant expression changes at different developmental stages, such as miR162a, whose expression remained below a TPM value of 1.

To understand the extent of sequence variation among *C. fortunei* miRNAs, we predicted novel miRNA sequences. A total of 145 unvalidated new miRNAs were predicted in this study. We named newly predicted miRNAs and numbered them in descending order according to the prediction evaluation score of the miRDeep2 software; their information is shown in Supplementary Table 4. In addition, we used the program RNAfold to predict the secondary structure of the top 5 novel miRNA precursors (Supplementary Figure 2). Each sequence consists of a star sequence, a stem-loop structure, and a mature sequence; the mature sequence can either be located in the 5′ arm or in the 3′ arm.

### Analysis of Differentially Expressed miRNAs and Prediction and Functional Annotation of Target Genes

**Figure 5:**
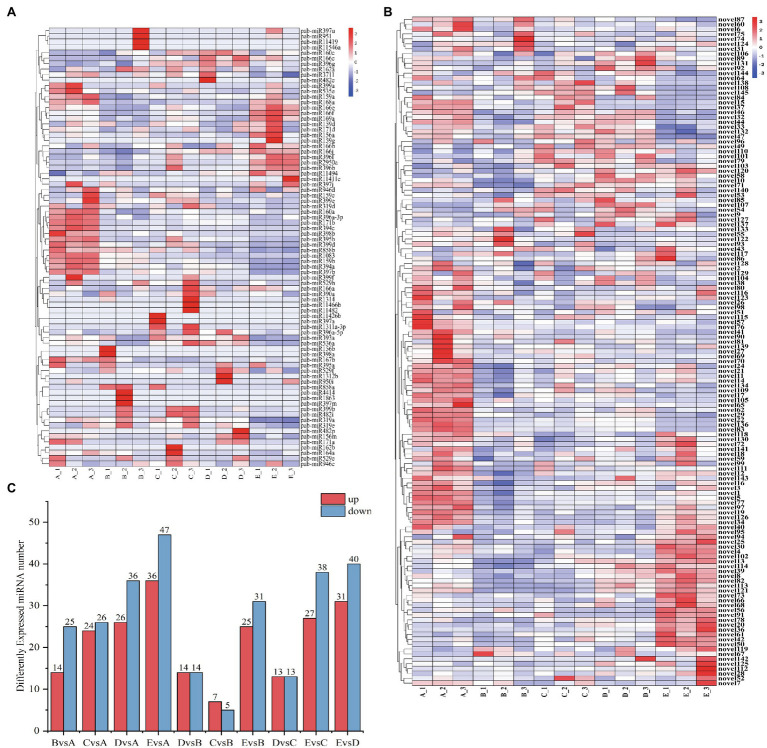
**(A)** Number of differentially expressed miRNAs when cross-comparing cambium developmental stages. The x-axis indicates which stages are being compared to each other, and the y-axis indicates the number of differentially expressed miRNAs within each comparison. **(B)** Clustering heatmap of differentially expressed novel miRNAs. The x-axis presents the sampled stages (A, B, C, D, and E; see “Materials and Methods”) and the biological replicate number. The miRNAs are shown on the y-axis. **(C)** Clustering heatmap of differentially expressed known miRNAs.

To understand the spatiotemporal expression patterns of miRNAs at successive stages of cambium development, we carried out differential expression analysis. We compared 5 stages, using |log2foldChange|>1 and a value of *p* <0.05 as filtering criteria (Figure 5A). In general, the number of expressed miRNAs during the different stages of cambium development is different. Differentially expressed genes were selected to produce a clustered heat map of their expression levels, showing each biological replicate from the individual stages A_1, 2, and 3 (Figures 5B,C). Based on the clustering results of the differentially expressed miRNAs and their expression across the various stages, these miRNAs could be roughly divided into three separate categories: Category 1, including miR858b, miR194c, miR159a, novel111, and novel77, was highly expressed during the A stage, and then, the expression levels decreased. Category 2, including miR319a, miR166e, miR397a, novel110, and novel145, was highly expressed in stages B, C, and D and expressed at low levels in stages A and E. Category 3, including miR166f, miR396f, miR11494, novel4, and novel82, was highly expressed in stage D.

**Figure 6:**
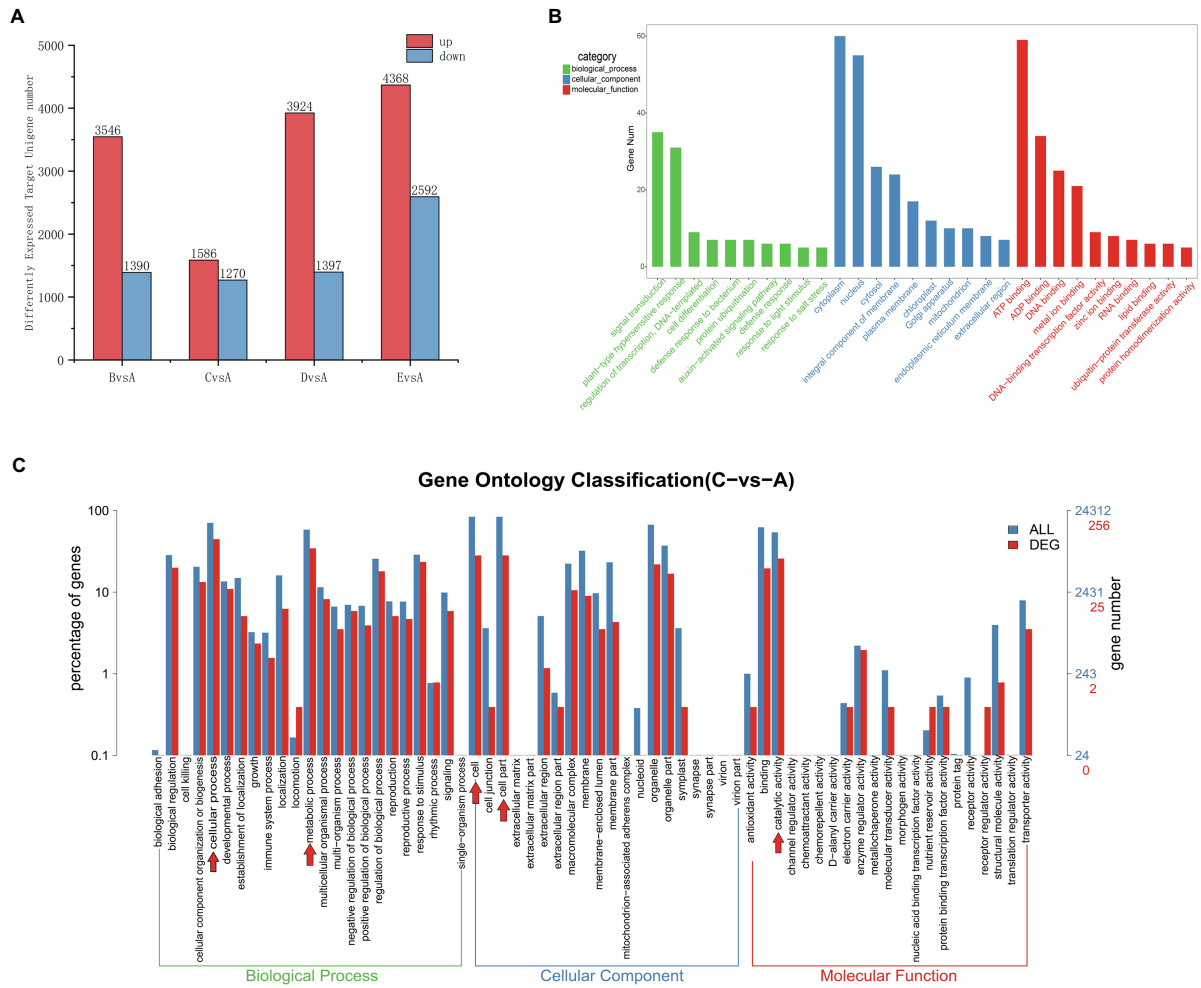
**(A)** Distribution of the number of differentially expressed target unigenes. The x-axis indicates which stage is being compared to the A stage, and the y-axis indicates the number of differentially expressed target genes. **(B)** Top 30 GO enrichment categories of the target unigenes (stage CvsA). The x-axis is the name of the GO entry, and the y-axis is the number of genes enriched in the GO entry. **(C)** Differently expressed miRNA target genes (red) shown in a GO Level2 horizontal distribution comparison plot (stage CvsA). For comparison, all target genes are plotted in blue. Red arrows indicate the 5 GO term subcategories with the highest number of DEGs for a particular stage comparison. The x-axis presents the different GO functional classifications, and the y-axes on the left and right represent the percentage and absolute number of unigenes classified to each GO category, respectively (stage CvsA).

The actions of miRNAs rely on complementary base pairing with target mRNA to form the RNA-induced silencing complex (RISC). The role of an miRNA is reflected by the function of its corresponding target genes; therefore, understanding the regulatory role of differentially expressed miRNAs is greatly aided by the prediction of their target genes. To determine the number of differentially expressed target genes at each stage, we compared expression in stage A to the remaining four stages (Figure 6A). We detected a higher number of upregulated and downregulated target genes in the B, D, and E stages in comparison with the A stage. Most target genes were differentially expressed in stage E (4368 upregulated and 2592 downregulated target genes), while the fewest target genes were differentially expressed in stage C (1586 upregulated and 1270 downregulated target genes). Among the samples from the E stage, we detected higher numbers of both upregulated and downregulated target genes compared to numbers in the B, C, and D stages.

To identify target genes for the miRNAs, we performed a target gene GO functional enrichment analysis, and we chose C vs. A (June vs. April) as an example to explain miRNA functionality. We performed a GO functional enrichment analysis on the differential target genes, which were classified into three separate categories: biological processes, cellular components, and molecular functions (Figure 6B). The most represented biological processes were signal transduction (GO:0007165), plant-type hypersensitive response (GO:0009626), DNA-based templates for transcriptional regulation (GO:0009626, GO:0006355), and cell differentiation (GO:0030154). The cellular components were nucleus (GO:0005634), cytoplasm (GO:0005737), and integral component of membrane (GO:0016021). The molecular functions were ATP binding (GO:0005524), ADP binding (GO:0043531), metal ion binding (GO:0046872), DNA binding (GO: 0003677), and DNA-binding-related transcription factor activity (GO:0003700). We plotted the GO term distribution for differentially expressed miRNA target genes (to estimate the degree of differential expression among target genes) for comparisons between each individual stage (Figure 6C). We plotted a total of 24,312 unigenes and 256 differentially expressed unigenes detected in the C-A stage comparison; the 5 most prevalent GO term subcategories of these genes were cellular process, metabolic process, cell, cellular part, and catalytic activity, accounting for 17.39, 13.42, 10.98, 10.98, 10.98, and 10.07%, respectively. To further study the functions of all differentially expressed miRNA target genes, we performed KEGG and GO analysis and found that most of the target genes were mainly associated with the metabolic pathway, DNA replication, plant hormone signal transduction, homologous recombination, and protein processing in the endoplasmic reticulum and were highly significant in the KEGG pathway (Supplementary Figures 3A,B), which may be closely related to cambial development. In the GO functional annotation, most genes were mainly concentrated in cellular process, single-organism process, cell, cell part, binding, and catalytic activity components (Supplementary Figure 3C).

### Validation of Gene Expression by qRT-PCR

**Figure 7:**
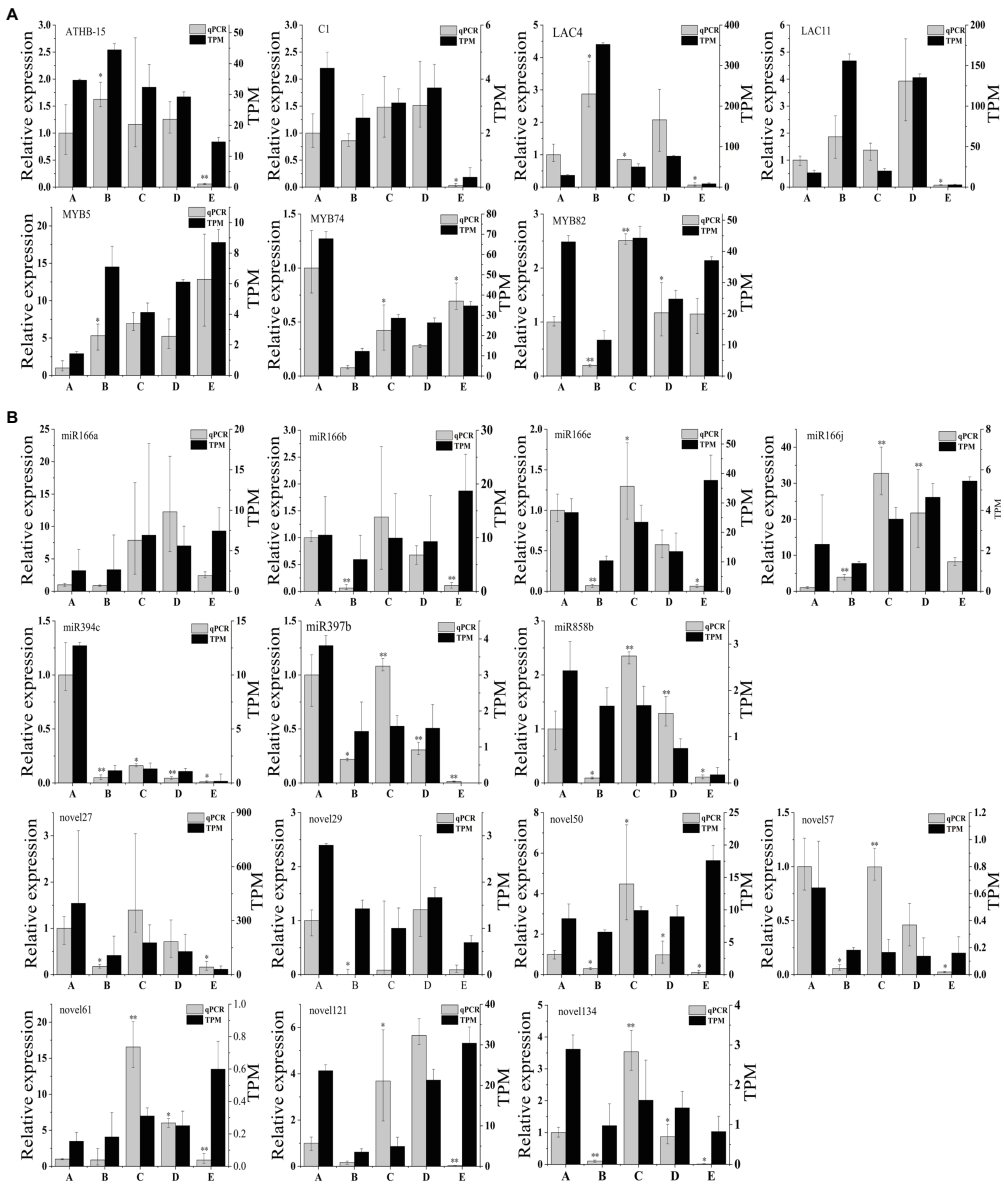
**(A)** qRT-PCR validation of genes in *C. fortunei*. **(B)** qRT-PCR validation of miRNAs in *C. fortunei*. The x-axis presents the 5 stages, and the y-axes on the left and right represent the relative expression and TPM values of genes or miRNAs, respectively; *n*=3, ^*^p<0.05, ^**^p<0.01.

To verify the RNA-seq results, we selected seven target genes for validation using quantitative real-time PCR (qRT-PCR), and *UBI* was selected as the best internal reference gene. We chose seven candidate genes related to vascular cambium development (*ATHB-15*:KF931396.1, *C1*:NM_129662.4, *LAC4*:AF132119.1, *LAC11*:AB762663.2, *MYB5*:KU131219.1, *MYB74*:KX887329.1, and *MYB82*:XM_014648707.2) for expression quantification *via* qRT-PCR assays (Figure 7A). We then compared the qRT-PCR results with TPM values obtained by sequencing during the different stages. We found that *ATHB-15*, *LAC4*, and *LAC11* were highly expressed in stage B and expressed at low levels in stage E. *C1* and *MYB82* were highly expressed in stage C; presumably, these genes may be involved in the development of the vascular cambium. *MYB74* was highly expressed in stage A and expressed at low levels in stage B, which may be involved in the break dormancy of the vascular cambium; however, the expression pattern of *MYB5* was different from that of *MYB82* and *MYB74*, which were highly expressed in stage E and expressed at low levels in stage A, possibly promoting the dormancy of the vascular cambium. Overall, the expression trends of these seven genes were consistent with the TPM value trends, showing that the transcriptome results are reliable.

We also chose seven known miRNAs (miR166a, miR166b, miR166e, miR166j, miR394c, miR397b, and miR858b) and seven novel miRNAs (novel27, novel29, novel50, novel57, novel61, novel121, and novel134) for expression quantification through qRT-PCR assays (Figure 7B). U6 was selected as the best internal reference gene. In the miRNA sequencing results, the target genes of these seven known miRNAs were all associated with vascular cambium development, and qRT-PCR results indicated that miR166a, b, e, and j expression decreased during vascular cambium B stage, rapidly increased during the C stage and decreased again during the E stage. We hypothesized that miR166 was involved in vascular cambium development. MiR394c is highly expressed during the A stage and presumably associated with stress resistance during dormancy in *C. fortunei*. miR397b and miR858b expression increased rapidly during the C stage and decreased during the E stage. We hypothesize that they are also involved in vascular cambium development. Comparison of the qRT-PCR results with the TPM values of known miRNAs and novel miRNAs revealed approximately the same trend, showing that the transcriptome results are highly reliable. However, miR166a, b, e, and j, novel50, novel61, and novel121 showed opposite trends at the E stage, which requires further investigation.

### Correlation Between miRNAs and Target Genes and Mapping of the Regulatory Network

**Figure 8:**
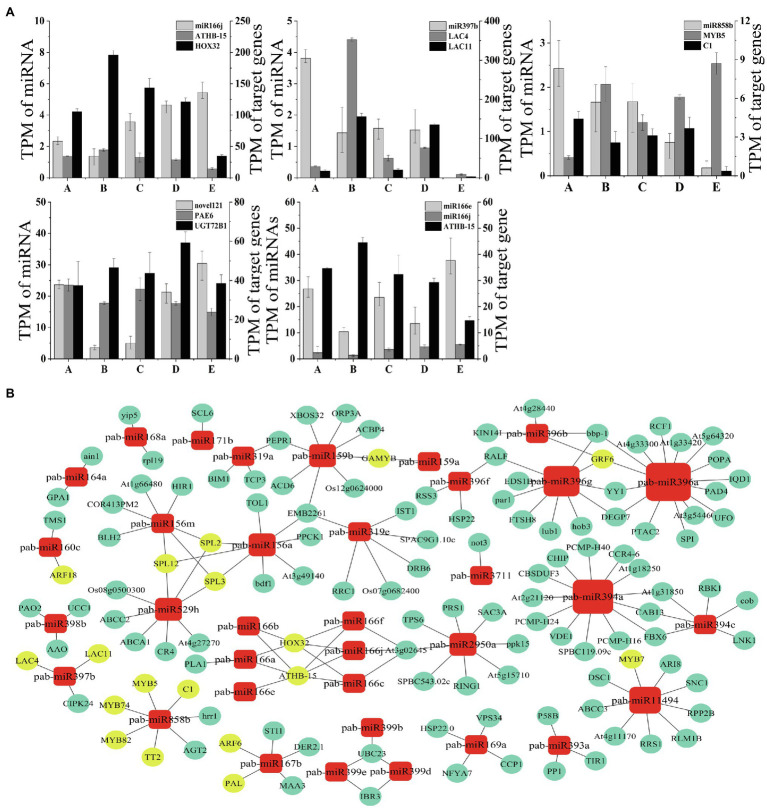
**(A)** Correlations of miRNA and target gene expression levels during *C. fortunei* vascular cambium development. The x-axis presents the five stages, and the y-axes on the left and right present the TPM values of the miRNAs and their target genes, respectively. **(B)** MicroRNA regulatory network. The nodes represent single miRNAs and target genes. Circles represent target genes, and squares represent miRNAs; the larger the node is, the greater the number of miRNA target gene pairs is.

To further verify and analyze the dynamic correlation in expression between miRNAs and their corresponding target genes, we selected four known miRNAs related to vascular cambium development and one novel miRNA and compared their expression with the expression of their target genes during vascular cambium development (Figure 8A). The miR166j and miR858b showed significant negative correlations with those of the target genes *ATHB-15*, *HOX32*, and *MYB5* in five stages, while the miR397b level had negative correlations with those of *LAC4* and *LAC11* in the A, B, C, and D stages. Novel121 also had negative regulatory effects on its target genes. We found that the miRNA expression was significantly negatively correlated with target gene expression in most stages; however, we did detect a positive correlation at individual stages, possibly due to the involvement of other regulatory mechanisms, which requires further experimental confirmation.

We used Cytoscape (version 3.5.1) to map the regulatory network and to determine the regulatory relationship between miRNAs and target genes. We selected known miRNAs that show dynamic expression during the *C. fortunei* vascular cambium development stage to predict and visualize a miRNA regulatory network diagram using Cytoscape software (Figure 8B). Yellow circles in the figure represent common transcription factors associated with vascular cambium growth and development, such as MYB, HD-ZIP, LAC, ARF, and GRF. We found that miR396a had the highest number of target genes involved in regulation, and miR3711 and miR171b had only one paired target gene. Target genes may be regulated by one or more miRNAs and by different members of the same miRNA family; for example, SPL is regulated by miR156a, miR156m, and miR529h. GAMYB, HOX32, ATHB-15, and GRF6 are then coregulated by different members of the same miRNA family. The results show that the relationship between miRNAs and target genes is not simple, and there is a complex regulatory network between them.

We predicted the partial miRNA target gene cleavage sites by using psRNATarget (Dai et al., 2018), and the miRNA was found to match the target gene exactly between the 10th and 11th base pair sites, which meets the requirements of miRNA-mediated target gene cleavage. Then, according to the plots obtained from the degradome sequencing data, some miRNAs were found to have potential target sites and a category ≤2 (Supplementary Figure 4). For example, miR159 cleaved *GAMYB* at site 805, which limited its function, while miR166f and miR166j cleaved *HD-ZIP* at sites 1350 and 1985.Identification of the specific cut site requires further experiments.

## Discussion

### Histomorphological Observation of the *C. fortunei* Vascular Cambium

*C. fortunei* is an important industrial tree species, and its vascular cambium exhibits periodic activity (Kentaro et al., 2014). In this study, we observed the number and thickness of vascular cambium cell layers in different stages and found increases in cambium cell division, cell layer number, and the cell thickness in stage A compared with stage E. We speculated that the cambium entered the germination stage from the dormant stage. In stage C, the number of cell layers peaks, cell division is vigorous, and the cambium enters the active phase. The gradual decrease in the number of cell layers in stages D and E may occur because the cambium enters the maturation stage and prepares for the dormant stage.

### Transcriptome Sequencing of *C. fortunei* Vascular Cambium

To better elucidate the functions of genes and miRNAs expressed in the vascular cambium at different developmental stages, we performed transcriptome sequencing and miRNA sequencing. There were a large number of DEGs identified during vascular cambium development, and we functionally annotated these DEGs and found that, similar to our previous studies, a large number of them were annotated to phenylpropanoid biosynthesis, plant hormone signal transduction and flavonoid biosynthesis (Cao et al., 2019). These processes are involved in plant secondary wall synthesis and lignin synthesis and play an important role in the development of vascular cambium. We constructed a total of 15 miRNA libraries, sequenced 60 known miRNAs, and predicted 145 unknown novel miRNAs. By analysis of the miRNA length, we found that 21nt was the most frequent class of miRNA, accounting for 33.1% of the total filtered reads. Previous studies have found that 21nt miRNAs are involved in mRNA degradation or translation inhibition, resulting in posttranscriptional silencing of genes, while 24nt miRNAs are mainly involved in the modification of DNA and histones, resulting in posttranscriptional silencing (Hamilton et al., 2002; Voinnet, 2009).

To annotate the known miRNAs we identified, we compared the filtered reads to the miRBase database. A total of 80 conserved miRNA sequences belonging to 32 miRNA families were obtained. The families with the most abundant expression were miR396, miR159, and miR319, while the families with the least abundant expression were miR946, miR399, and miR529. miR159 (Millar et al., 2019), miR160 (Ding et al., 2017; Wójcik et al., 2017), and miR166 (Yan et al., 2016; Yang et al., 2020b) are known to be mainly involved in hormone signal transduction, while miR166 (Zhou et al., 2015), miR167 (Cai et al., 2019), and miR396 (Debernardi et al., 2014) are mainly involved in growth and development, and miR394 (Song et al., 2013), miR156 (Chen et al., 2020b), and miR395 (Ai et al., 2016) are mainly involved in stress response regulation.

### Regulation of Hormone Expression by miRNAs in Vascular Cambium

The growth and development of the vascular cambium are closely related to plant hormones. Two plant hormones strongly involved in plant development and dormancy are ABA and GA. During phases of vigorous plant growth, the level of ABA decreases, and the level of GA increases (Liu et al., 2011). miR166 is involved in ABA signal transduction in plants and expressed at low levels during somatic embryo maturation (Yan et al., 2016; Yang et al., 2020b). These findings are consistent with the conclusion that ABA is a key factor for somatic embryo maturation, as low miR166 levels would lead to high levels of ABA. In this experiment, miR166 showed high expression during the A stage, which led to a decrease in ABA content in the vascular cambium of *C. fortunei* and promoted the transition of the vascular cambium to the active phase. In *Arabidopsis thaliana*, miR159 also regulates ABA signal transduction. We detected the highest miR159 expression in *C. fortunei* cambium during stage A dormancy release, when *MYB33* and *MYB101*were inhibited and ABA synthesis was decreased (Supplementary Table 3). It is hypothesized that this regulatory mechanism is crucial for dormancy release in the vascular cambium (Wang et al., 2017). miR160 is involved in the formation, accumulation and transport of plant IAA and causes auxin accumulation, thereby inducing the autonomous formation of somatic embryos in Arabidopsis explants (Ding et al., 2017; Wójcik et al., 2017). The involvement of miR160 in mediating the transport of growth hormone is also essential for vascular cambium activity, and high expression of miR160 during the A stage caused the accumulation of IAA, which promoted the development of the formation layer (Supplementary Table 3).

### Regulation of Transcription Factors by miRNAs in the Vascular Cambium

The regulatory relationship of miRNA transcription factors plays an important role during cambium development and plant growth. For example, miR166 negatively regulates organ polarity and cell development (Zhou et al., 2015). We detected the expression of three miR166 family members in *C. fortunei* cambium: miR166a, b, and e. miR166a showed high expression during the C and D stages, while miR166b and e exhibited a more fluctuating expression pattern (Supplementary Table 3). In this study, the target genes of miR166 were mainly *HOX32* and *ATHB-15*, which were classified as members of the HD-ZIP III transcription factor family (Figure 8). Additionally, it was found that miR166 had a negative regulatory effect on the HD-ZIPIII transcription factor PtaHB1 in *Populus euphratica*, which in turn had an effect on wood formation density (Ko et al., 2006). Therefore, we hypothesize that miR166 affects important factors in vascular cambium development when differentially expressed in growth stages. miR159-*GAMYB* plays an important regulatory role in plant growth (Reyes and Chua, 2007) and has an important effect on plant growth (Zheng et al., 2020; Figure 8B). In this study, we found that the high abundance of miR159 may effectively inhibit the effect of GAMYB on vascular cambium development in *C. fortunei*. The target genes of miR396 are GRFs, a class of regulators that regulate plant growth and development (Figure 8B). In *Arabidopsis thaliana*, the miR396 family regulates a large number of GRFs, promoting plant growth (Debernardi et al., 2014). Furthermore, miR396 is a temperature-sensitive gene, and its expression is dramatically inhibited at low temperature. In *Cymbidium goeringii* (Rchb.f.), miR396 increases photosynthetic efficiency, leaf growth, and development during the reproductive growth stage and reduces the stomatal conductance of leaves (Xu et al., 2020). Here, we found miR396 to be mainly expressed at the D and E stages, which occur during the main growth season at higher temperatures, consistent with the findings reported in *Arabidopsis thaliana*. We speculate that low expression of miR396 during developmental stages leads to elevated expression of GRFs and facilitates the development of the vascular cambium and that the high expression of miR396 at the mature stage may inhibit GRF levels in preparation for subsequent dormancy. miR394 mainly targets F-box family proteins, and high expression of miR394 decreases F-box protein expression levels and enhances stress resistance (Song et al., 2013). Here, we found that miR394 was mainly expressed during stage A (Supplementary Table 3); this expression may be necessary to increase stress resistance during a time of the year when plants have less access to water and to improve their chances of surviving the cold winter, a time that is not conducive to growth.

## Conclusion

*C. fortunei* is a tree species that has a beautiful shape, excellent wood properties and wood that is easily processed and produced. In this study, we carried out transcriptome sequencing and miRNA functional analysis of the vascular cambium in different growth stages in *C. fortunei*. We identified the DEGs and miRNAs that function in the development of the vascular cambium, analyzed the spatiotemporal expression patterns of related miRNAs and target genes, analyzed the correlations in expression between some miRNAs and their target genes in different growth stages, and identified the target cleavage sites of some miRNAs by degradome sequencing. The purpose of this study was to provide a theoretical basis for further elucidating the mechanisms underlying the growth and development of *C. fortunei* vascular cambium cells and the molecular mechanism underlying wood formation.

## Data Availability Statement

The datasets presented in this study can be found in online repositories. The names of the repository/repositories and accession number(s) can be found at: https://www.ncbi.nlm.nih.gov/, PRJNA698260.

## Author Contributions

JX: conceptualization, funding acquisition, supervision, and project administration. HH, ZG, JY, YZ, and JC: formal analysis. HH: data curation. HH and ZG: writing – original draft. HH and YZ: writing – review and editing. All authors have read and agreed to the published version of the manuscript.

## Conflict of Interest

The authors declare that the research was conducted in the absence of any commercial or financial relationships that could be construed as a potential conflict of interests.

## Publisher’s Note

All claims expressed in this article are solely those of the authors and do not necessarily represent those of their affiliated organizations, or those of the publisher, the editors and the reviewers. Any product that may be evaluated in this article, or claim that may be made by its manufacturer, is not guaranteed or endorsed by the publisher.

## Footnotes

^1^ http://www.sanger.ac.uk

^2^ https://www.mirbase.org/index.shtml

^3^ http://rna.tbi.univie.ac.at/cgi-bin/RNAWebSuite/RNAfold.cgi
