# Supplementary material for: Transcriptome and microRNA Sequencing Identified miRNAs and Target Genes in Different Developmental Stages of the Vascular Cambium in Cryptomeria fortunei Hooibrenk
Source: Front Plant Sci. 2021 Nov 18;12:751771. doi: 10.3389/fpls.2021.751771 (PMC8638621; doi:10.3389/fpls.2021.751771)
Supplement: Supplementary file 1 [file Data_Sheet_1.zip › Supplementary Figure 1.docx]

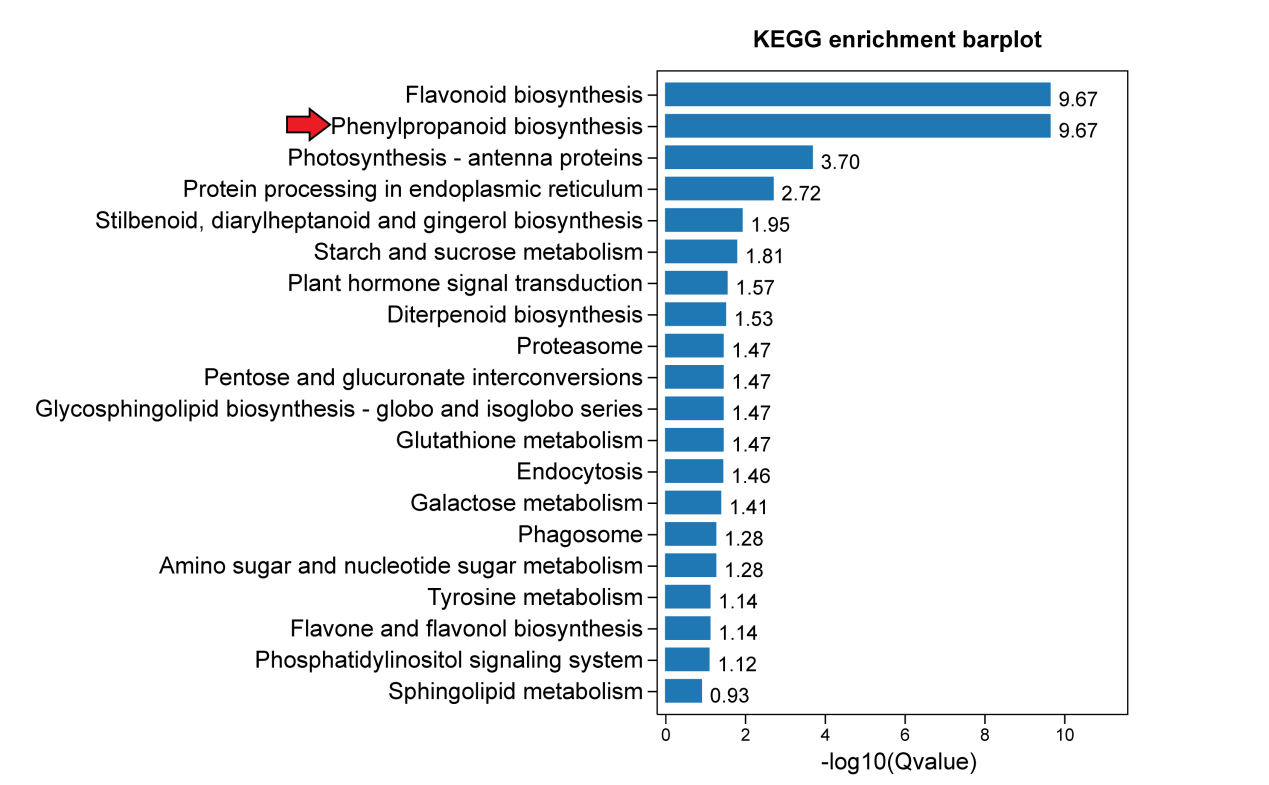


Supplementary Figure 1. KEGG enrichment barplot of DEGs. A larger number in the figure means that the enrichment is more significant.
