# Supplementary material for: Transcriptome and microRNA Sequencing Identified miRNAs and Target Genes in Different Developmental Stages of the Vascular Cambium in Cryptomeria fortunei Hooibrenk
Source: Front Plant Sci. 2021 Nov 18;12:751771. doi: 10.3389/fpls.2021.751771 (PMC8638621; doi:10.3389/fpls.2021.751771)
Supplement: Supplementary file 1 [file Data_Sheet_1.zip › Supplementary Figure 2.docx]

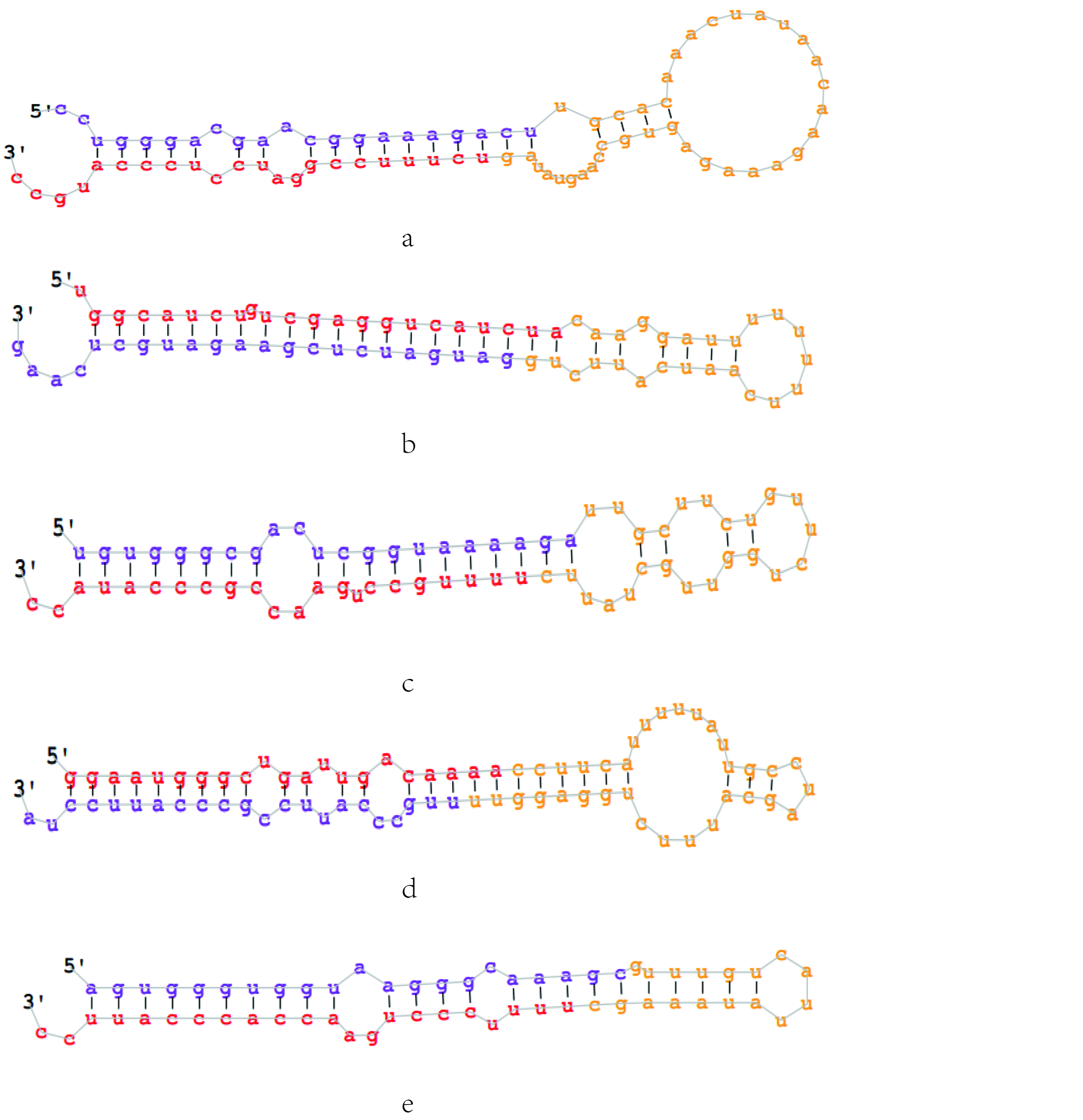


Supplementary Figure 2. Prediction map of the secondary structure of miRNA precursors. Indicated are the mature sequence (red), the stem ring structure (yellow) and the STAR sequence (purple); a:novel1; b:novel2; c:novel3; d:novel4; e:novel5.
