# Supplementary material for: Transcriptome and microRNA Sequencing Identified miRNAs and Target Genes in Different Developmental Stages of the Vascular Cambium in Cryptomeria fortunei Hooibrenk
Source: Front Plant Sci. 2021 Nov 18;12:751771. doi: 10.3389/fpls.2021.751771 (PMC8638621; doi:10.3389/fpls.2021.751771)
Supplement: Supplementary file 1 [file Data_Sheet_1.zip › Supplementary Figure 3.docx]

Supplementary Figure 3. (A) KEGG analysis of differentially expressed target genes. The x-axis shows the number of differentially expressed target genes annotated to each pathway; the y-axis indicates the name of the pathway, and the number on the right side of the column indicates the number of differentially expressed target genes annotated to each pathway. (B) Top 20 enrichment pathways of differentially expressed target genes. The x- and y-axes represent the enrichment factor and the pathway term, respectively. The colors and sizes of the dots represent the significance and the number of differentially expressed target genes. (C) GO annotation of differentially expressed target genes. The x-axis presents the different GO functional classifications, and the y-axes on the left and right represent the number of and percentage of target genes classified to each GO category, respectively
