# Supplementary figures and images for: Transcriptome and microRNA Sequencing Identified miRNAs and Target Genes in Different Developmental Stages of the Vascular Cambium in Cryptomeria fortunei Hooibrenk
Source: Front Plant Sci. 2021 Nov 18;12:751771. doi: 10.3389/fpls.2021.751771 (PMC8638621; doi:10.3389/fpls.2021.751771)

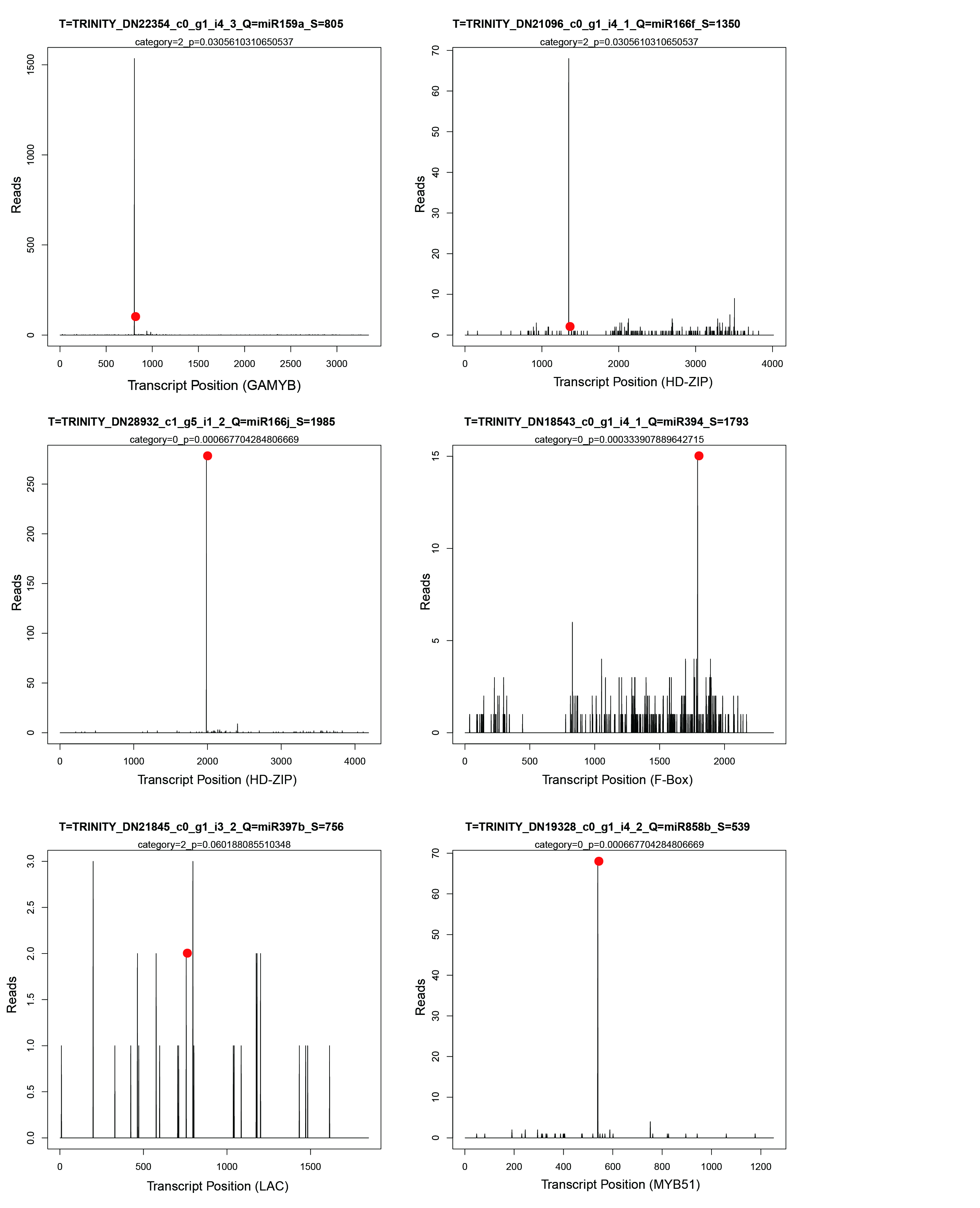


Supplementary Figure 4. Target plots of miRNA targets confirmed by degradome sequencing.

Supplement: Supplementary file 1 [file Data_Sheet_1.zip › Supplementary Figure 4.docx]
