# Supplementary material for: Transcriptome and microRNA Sequencing Identified miRNAs and Target Genes in Different Developmental Stages of the Vascular Cambium in Cryptomeria fortunei Hooibrenk
Source: Front Plant Sci. 2021 Nov 18;12:751771. doi: 10.3389/fpls.2021.751771 (PMC8638621; doi:10.3389/fpls.2021.751771)
Supplement: Supplementary file 1 [file Data_Sheet_1.zip › Supplementary Tables.doc]

**Supplementary Table 1. All primers used in this test**

| Primer name | Primer sequence(5’-3’) | Primer length(bp) |
| --- | --- | --- |
| RT-*ATHB*-15-F | CGGGAAGTATGTTCGTTA | 18 |
| RT-*ATHB*-15-R | TTGCTTGGGTTCTATGTT | 18 |
| RT-*LAC4*-F | CACAGTGAGTCTTGGGTTTA | 20 |
| RT-*LAC4*-R | TCCAGCGGTGTTTCTTTC | 18 |
| RT-*LAC11*-F | CAGGTGGGTGGACTGCTA | 18 |
| RT-*LAC11*-R | CTCAGCAGGCATACTCCA | 20 |
| RT-*MYB5*-F | TGAACTATCTTCGTCCCG | 18 |
| RT-*MYB5*-R | TGTTGACAGGTAGGTGGTA | 19 |
| RT-*MYB82*-F | ACAAACCCACTCATTACC | 18 |
| RT-*MYB82*-R | CCATTATTATTGCTGCTACT | 20 |
| RT-*MYB74*-F | GGTAACAGGTGGGCTCAAAT | 20 |
| RT-*MYB74*-R | TGACATAGAGGCTTGTGGGT | 20 |
| RT-*C1*-F | GGGAACGGTAACGGAATCAC | 20 |
| RT-*C1*-R | GCTCTAATGGCGGCATCAAG | 20 |
| *GAPDH*-F | TTGGCATCGTTGAGGGTC | 18 |
| *GAPDH*-R | CAGTGGGAACACGGAAAGC | 19 |
| *18sRNA*-F | AATCCGACACAGAGACGAATC | 21 |
| *18sRNA*-R | ACCCACACCCAATATCAAGTAG | 22 |
| *UBI/UBQ*-F | CGTTAAAGCCAAGATCCAGGACAA | 24 |
| *UBI/UBQ*-R | TCCATCCTCAAGCTGTTTCCC | 21 |
| pab-miR166a-FS | GTCGTATCCAGTGCAGGGTCCGAGGTATTCGCACTGGATACGACGGGTGG | 50 |
| pab-miR166b-FS | GTCGTATCCAGTGCAGGGTCCGAGGTATTCGCACTGGATACGACGGTGGC | 50 |
| pab-miR166e-FS | GTCGTATCCAGTGCAGGGTCCGAGGTATTCGCACTGGATACGACGGTGGC | 50 |
| pab--miR166j-FS | GTCGTATCCAGTGCAGGGTCCGAGGTATTCGCACTGGATACGACGGGGAA | 50 |
| pab-miR394c-FS | GTCGTATCCAGTGCAGGGTCCGAGGTATTCGCACTGGATACGACGGGGTG | 50 |
| pab-miR397b-FS | GTCGTATCCAGTGCAGGGTCCGAGGTATTCGCACTGGATACGACCGTCAA | 50 |
| pab-miR858b-FS | GTCGTATCCAGTGCAGGGTCCGAGGTATTCGCACTGGATACGACCAAGGT | 50 |
| pab-miR166a-RT | GCGCGCGCGGACCAGG | 16 |
| pab-miR166b-RT | CGCGCGCGCGGACCAG | 16 |
| pab-miR166e-RT | GCGCGCGCCGGACCAG | 16 |
| Pab-miR166j-RT | CGCCGGACCAGGCTTCA | 17 |
| pab-miR394c-RT | GCGCGCGCGGGCACGC | 16 |
| Pab-miR397b-RT | GCGTCATTGAGTGCAGCG | 18 |
| Pab-miR858b-RT | CGCGTTCGTTGTCTGTTCG | 19 |
| novel121mature-RT | GCGCGCGACCCCAAAG | 16 |
| novel134mature-RT | GCGCGCGGGGGAAGGG | 16 |
| novel27mature-RT | CGCGCGCGCGGCACGC | 16 |
| novel29mature-RT | CGCGCGCGCGGCCGGC | 16 |
| novel50mature-RT | GCGCGCGCAACACGAA | 16 |
| novel57mature-RT | CGCGCGCGCAGGGCAC | 16 |
| novel61mature-RT | GCGCGCGCGAAGACCA | 16 |
| mQ Primer | AGTGCAGGGTCCGAGGTATT | 20 |
| *U6*-F | CTCGCTTCGGCAGCACA | 17 |
| *U6*-R | AACGCTTCACGAATTTGCGT | 20 |

**Supplementary Table 2. Structure of miRNA families**

| miRNA family ID | Member miRNAs | miRNA number | |
| --- | --- | --- | --- |
| miR1311 | novel115_mature; novel84_star; pab-miR1311a-3p | 3 |  |
| miR1312 | pab-miR1312b | 1 |  |
| miR1314 | pab-miR1314 | 1 |  |
| miR156 | novel46_mature; pab-miR156a>pab-miR156l; pab-miR156b>pab-miR156c>pab-miR156d>pab-miR156e>pab-miR156f>pab-miR156g>pab-miR156h>pab-miR156i>pab-miR156j>pab-miR156k; pab-miR156m>pab-miR156n>pab-miR156p>pab-miR156q>pab-miR156r>pab-miR156s>pab-miR156t>pab-miR156u>pab-miR156v>pab-miR156w | 4 |  |
| miR159 | pab-miR159a; pab-miR159b>pab-miR159c; pab-miR159d; pab-miR159e>pab-miR159j>pab-miR159k; pab-miR159g>pab-miR159h>pab-miR159i; pab-miR319d>pab-miR319h; pab-miR4414 | 7 |  |
| miR160 | novel29_mature; novel29_star; pab-miR160a>pab-miR160b>pab-miR160d; pab-miR160c>pab-miR160e>pab-miR160f | 4 |  |
| miR162 | pab-miR162a; pab-miR162b>pab-miR162c | 2 |  |
| miR164 | novel24_mature; pab-miR164a | 2 |  |
| miR166 | pab-miR166a; pab-miR166b; pab-miR166c>pab-miR166d; pab-miR166e; pab-miR166f>pab-miR166g>pab-miR166h>pab-miR166i; pab-miR166j | 6 |  |
| miR167 | pab-miR167b | 1 |  |
| miR168 | pab-miR168a | 1 |  |
| miR169 | pab-miR169a>pab-miR169b>pab-miR169c>pab-miR169d>pab-miR169e | 1 |  |
| miR171 | novel136_mature; novel22_mature>novel23_mature;novel22_star>novel23_star; novel76_mature; novel76_star; pab-miR171a; pab-miR171b>pab-miR171c; pab-miR171d>pab-miR171e | 8 |  |
| miR2118 | novel19_mature | 1 |  |
| miR2950 | novel73_mature; pab-miR2950a>pab-miR2950b | 2 |  |
| miR319 | pab-miR319a>pab-miR319b>pab-miR319c>pab-miR319f>pab-miR319g>pab-miR319i>pab-miR319k>pab-miR319l>pab-miR319m>pab-miR319n; pab-miR319e | 2 |  |
| miR390 | pab-miR390a>pab-miR390b | 1 |  |
| miR393 | pab-miR393a>pab-miR393b>pab-miR393c>pab-miR393d | 1 |  |
| miR394 | novel26_mature;novel27_mature;pab-miR394a>pab-miR394b; pab-miR394c | 4 |  |
| miR395 | pab-miR395b>pab-miR395c>pab-miR395d>pab-miR395e>pab-miR395g | 1 |  |
| miR396 | novel15_mature; pab-miR396a-3p>pab-miR396d>pab-miR396e; pab-miR396a-5p; pab-miR396b>pab-miR396c>pab-miR396i>pab-miR396j>pab-miR396k>pab-miR396l>pab-miR396m>pab-miR396n>pab-miR396o>pab-miR396p>pab-miR396q>pab-miR396r>pab-miR396s>pab-miR396t; pab-miR396f;pab-miR396g>pab-miR396h | 6 |  |
| miR397 | pab-miR397a>pab-miR397c>pab-miR397d>pab-miR397f>pab-miR397g>pab-miR397h; pab-miR397b; pab-miR397j>pab-miR397k>pab-miR397l;pab-miR397m>pab-miR397n>pab-miR397o>pab-miR397p>pab-miR397q>pab-miR397r>pab-miR397s>pab-miR397t | 4 |  |
| miR398 | pab-miR398a; pab-miR398b | 2 |  |
| miR399 | novel37_mature; novel37_star; novel55_mature;novel55_star; novel69_mature; pab-miR399a; pab-miR399b>pab-miR399c; pab-miR399d; pab-miR399e; pab-miR399f>pab-miR399g | 10 |  |
| miR477 | novel121_mature; novel39_mature | 2 |  |
| miR482 | novel21_mature; novel30_star;novel4_star; pab-miR482c;pab-miR482i | 5 |  |
| miR5037 | novel113_mature; novel25_mature | 2 |  |
| miR529 | pab-miR529e; pab-miR529f>pab-miR529g>pab-miR529i>pab-miR529k>pab-miR529l>pab-miR529m>pab-miR529n>pab-miR529o; pab-miR529h>pab-miR529j | 3 |  |
| miR535 | pab-miR535e>pab-miR535a>pab-miR535c>pab-miR535d | 1 |  |
| miR858 | pab-miR858a; pab-miR858b | 2 |  |
| miR946 | pab-miR946c>pab-miR946e;pab-miR946d | 2 |  |
| miR951 | pab-miR951 | 1 |  |

**Supplementary Table 3. Known miRNA expression statistics of clean reads**

| miRNAs | Reads count (TPM) | | | | | | | | | | | | | | | Length (bp) |
| --- | --- | --- | --- | --- | --- | --- | --- | --- | --- | --- | --- | --- | --- | --- | --- | --- |
|  | A_1 | A_2 | A_3 | B_1 | B_2 | B_3 | C_1 | C_2 | C_3 | D_1 | D_2 | D_3 | E_1 | E_2 | E_3 |  |
| pab-miR1083 | 1237.711 | 1444.310 | 1442.119 | 518.778 | 544.349 | 626.437 | 161.125 | 158.549 | 183.154 | 66.157 | 64.430 | 64.965 | 81.444 | 86.970 | 77.530 | 21 |
| pab-miR11494 | 0.170 | 0.449 | 0.314 | 0.670 | 0.661 | 0.396 | 0.392 | 0.491 | 0.432 | 0.613 | 0.522 | 0.602 | 0.912 | 0.672 | 0.750 | 21 |
| pab-miR156a/l | 0.988 | 1.650 | 1.259 | 0.939 | 0.955 | 1.654 | 0.588 | 0.655 | 0.504 | 1.157 | 0.783 | 0.376 | 3.227 | 4.231 | 1.188 | 20 |
| pab-miR156m/n/p/q/r/s/t/u/v/w | 0.898 | 0.900 | 0.734 | 0.403 | 0.294 | 0.331 | 0.327 | 0.819 | 0.216 | 0.408 | 1.175 | 1.882 | 0.070 | 0.470 | 0.000 | 21 |
| pab-miR159a | 135.518 | 160.321 | 184.841 | 67.766 | 57.895 | 87.327 | 105.173 | 98.930 | 87.903 | 101.142 | 109.931 | 102.003 | 113.644 | 151.241 | 78.969 | 21 |
| pab-miR159b/c | 2387.231 | 2893.194 | 2883.504 | 1367.330 | 1589.847 | 1718.089 | 1076.369 | 1207.137 | 1474.312 | 1109.972 | 1038.988 | 943.847 | 493.719 | 603.217 | 439.611 | 20 |
| pab-miR160a/b/d | 50.381 | 60.739 | 62.208 | 27.241 | 12.637 | 14.687 | 35.428 | 28.991 | 33.216 | 27.634 | 27.874 | 23.186 | 34.374 | 23.640 | 25.135 | 21 |
| pab-miR160c/e/f | 3.323 | 3.824 | 3.567 | 0.268 | 10.139 | 8.733 | 4.576 | 14.414 | 13.041 | 16.063 | 17.952 | 12.120 | 2.455 | 9.939 | 9.129 | 21 |
| pab-miR162a | 0.090 | 0.375 | 0.210 | 0.134 | 0.588 | 0.463 | 0.196 | 0.491 | 0.216 | 0.476 | 0.131 | 0.376 | 0.140 | 0.269 | 0.188 | 21 |
| pab-miR164a | 2.066 | 1.500 | 2.937 | 1.946 | 1.543 | 0.794 | 0.915 | 5.733 | 0.865 | 0.817 | 0.849 | 1.054 | 0.421 | 0.336 | 1.250 | 21 |
| pab-miR166a | 1.347 | 5.174 | 1.154 | 0.335 | 0.735 | 6.946 | 1.503 | 1.065 | 18.229 | 8.031 | 0.914 | 7.904 | 10.312 | 7.992 | 4.064 | 21 |
| pab-miR166b | 10.777 | 3.074 | 17.624 | 3.355 | 4.041 | 10.453 | 10.981 | 18.181 | 0.648 | 0.885 | 17.821 | 9.184 | 25.535 | 14.439 | 16.194 | 21 |
| pab-miR166e | 31.432 | 23.546 | 25.387 | 11.943 | 9.919 | 9.394 | 20.263 | 29.237 | 21.039 | 19.738 | 11.293 | 9.485 | 32.550 | 46.205 | 34.326 | 21 |
| pab-miR166f/g/h/i | 261.606 | 258.928 | 281.983 | 97.489 | 55.912 | 128.146 | 227.144 | 325.534 | 227.322 | 162.398 | 173.317 | 174.572 | 665.026 | 873.262 | 580.604 | 21 |
| pab-miR166j | 2.245 | 2.100 | 2.623 | 1.476 | 0.808 | 1.852 | 3.007 | 4.095 | 3.603 | 4.901 | 4.896 | 4.140 | 6.103 | 5.171 | 5.064 | 21 |
| pab-miR168a | 12.663 | 12.523 | 16.890 | 3.355 | 2.498 | 4.102 | 1.830 | 3.849 | 3.819 | 3.607 | 4.047 | 3.388 | 11.785 | 13.499 | 8.691 | 21 |
| pab-miR171b/c | 980.146 | 1127.343 | 1040.231 | 115.135 | 116.452 | 188.613 | 231.654 | 135.864 | 261.618 | 300.907 | 310.078 | 245.334 | 26.377 | 25.789 | 24.510 | 21 |
| pab-miR2950a/b | 14.369 | 12.973 | 17.309 | 15.767 | 6.245 | 18.855 | 19.806 | 54.051 | 16.428 | 17.492 | 18.735 | 22.207 | 59.137 | 76.225 | 72.904 | 21 |
| pab-miR319a/b/c/f/g/i/k/l/m/n | 825.140 | 1102.898 | 1014.634 | 856.401 | 1270.246 | 765.234 | 1070.094 | 855.069 | 1352.762 | 850.175 | 796.801 | 848.995 | 332.303 | 358.492 | 308.497 | 20 |
| pab-miR319d/h | 12.303 | 1.350 | 16.155 | 9.729 | 3.674 | 1.654 | 8.105 | 0.409 | 16.932 | 10.141 | 1.436 | 0.753 | 3.297 | 0.604 | 1.688 | 20 |
| pab-miR319e | 0.449 | 0.300 | 1.049 | 0.805 | 1.469 | 0.463 | 0.654 | 0.737 | 1.441 | 0.545 | 0.979 | 0.753 | 0.421 | 0.269 | 0.125 | 20 |
| pab-miR3711 | 5.478 | 7.424 | 8.183 | 4.562 | 4.629 | 7.013 | 5.295 | 7.698 | 5.836 | 9.937 | 5.549 | 5.119 | 3.157 | 4.634 | 1.626 | 20 |
| pab-miR390a/b | 0.449 | 0.300 | 0.000 | 0.738 | 0.147 | 0.331 | 0.261 | 0.491 | 1.081 | 0.681 | 0.261 | 0.301 | 0.351 | 0.336 | 0.063 | 21 |
| pab-miR393a/b/c/d | 0.539 | 0.750 | 0.525 | 0.805 | 0.073 | 0.066 | 1.111 | 0.655 | 0.721 | 0.545 | 0.653 | 1.129 | 0.000 | 0.134 | 0.063 | 22 |
| pab-miR394a/b | 383.563 | 555.273 | 469.447 | 211.886 | 277.354 | 187.356 | 222.503 | 117.438 | 244.182 | 245.912 | 208.829 | 175.852 | 29.603 | 35.393 | 31.262 | 20 |
| pab-miR394c | 10.507 | 15.147 | 12.484 | 1.006 | 1.249 | 1.191 | 1.896 | 0.328 | 1.729 | 0.885 | 1.763 | 0.602 | 0.070 | 0.067 | 0.375 | 20 |
| pab-miR395b/c/d/e/g | 18.590 | 20.171 | 22.869 | 3.892 | 3.012 | 3.639 | 3.203 | 18.590 | 6.341 | 7.623 | 6.528 | 6.474 | 2.455 | 3.089 | 1.376 | 21 |
| pab-miR396a-3p/d/e | 207.812 | 264.552 | 236.035 | 60.318 | 105.138 | 22.162 | 75.497 | 77.145 | 112.472 | 88.482 | 94.525 | 95.002 | 46.720 | 51.376 | 106.042 | 20 |
| pab-miR396a-5p | 1210.679 | 1585.284 | 912.667 | 1159.738 | 1410.797 | 1164.093 | 2340.401 | 1122.785 | 3126.591 | 1627.863 | 2908.788 | 1704.540 | 1356.498 | 1063.790 | 1467.766 | 21 |
| pab-miR396b/c/i/j/k/l/m/n/o/p/q/r/s/t | 696.807 | 632.359 | 803.567 | 629.217 | 321.952 | 807.972 | 743.791 | 2014.788 | 795.374 | 837.175 | 865.802 | 889.571 | 1435.768 | 1824.226 | 1825.594 | 21 |
| pab-miR396f | 2.245 | 2.250 | 4.721 | 2.818 | 1.396 | 4.300 | 3.595 | 7.452 | 3.747 | 4.016 | 3.199 | 4.743 | 5.472 | 9.201 | 8.628 | 21 |
| pab-miR396g/h | 10146.500 | 8166.827 | 9549.750 | 12375.010 | 6904.470 | 12135.850 | 16668.650 | 17286.790 | 16944.940 | 20261.140 | 16403.490 | 19414.050 | 11547.110 | 14623.420 | 11768.070 | 21 |
| pab-miR397b | 3.682 | 3.674 | 4.091 | 0.805 | 1.249 | 2.249 | 1.634 | 1.228 | 1.873 | 2.178 | 1.110 | 1.280 | 0.000 | 0.000 | 0.000 | 21 |
| pab-miR399b/c | 1.437 | 1.050 | 1.049 | 1.342 | 14.547 | 0.132 | 0.719 | 14.741 | 16.356 | 0.340 | 0.326 | 6.098 | 0.210 | 0.269 | 2.001 | 21 |
| pab-miR399d | 205.657 | 150.123 | 168.791 | 90.444 | 70.239 | 37.379 | 68.176 | 142.580 | 126.954 | 35.052 | 29.115 | 42.231 | 17.888 | 23.707 | 18.632 | 21 |
| pab-miR399e | 0.090 | 0.300 | 1.049 | 0.067 | 0.294 | 0.265 | 0.196 | 0.491 | 0.504 | 0.068 | 0.065 | 0.151 | 0.140 | 0.000 | 0.063 | 21 |
| pab-miR529f/g/i/k/l/m/n/o | 0.269 | 0.150 | 0.105 | 0.470 | 0.220 | 0.265 | 0.261 | 0.409 | 0.216 | 0.136 | 0.718 | 0.452 | 0.000 | 0.403 | 0.000 | 21 |
| pab-miR535a/c/d/e | 3.233 | 4.424 | 1.574 | 0.939 | 1.469 | 1.720 | 0.980 | 0.901 | 2.089 | 1.293 | 0.914 | 0.678 | 1.333 | 2.283 | 0.750 | 21 |
| pab-miR536a | 0.269 | 0.375 | 0.315 | 0.134 | 0.000 | 0.000 | 0.327 | 0.328 | 0.865 | 0.681 | 0.392 | 0.527 | 0.070 | 0.000 | 0.000 | 21 |
| pab-miR858b | 3.053 | 2.025 | 2.203 | 1.946 | 2.057 | 0.992 | 1.961 | 0.983 | 2.089 | 0.953 | 0.392 | 0.903 | 0.140 | 0.336 | 0.063 | 21 |
| pab-miR946c/e | 0.539 | 0.150 | 0.000 | 0.067 | 0.073 | 0.331 | 0.196 | 0.573 | 0.288 | 0.136 | 0.000 | 0.301 | 0.000 | 0.470 | 0.000 | 22 |
| pab-miR946d | 0.090 | 0.300 | 0.525 | 0.201 | 0.294 | 0.066 | 0.196 | 0.246 | 0.216 | 0.068 | 0.196 | 0.075 | 0.421 | 0.000 | 0.313 | 22 |

**Supplementary Table 4. Predicted miRNA information**

| Novel ID | miRDeep2 score | Consensus mature sequence | Precursor sequence information  (+: on positive chain; -: on negative chain) |
| --- | --- | --- | --- |
| novel1 | 2282.4 | ucuuuccggauccucccaugcc | TRINITY_DN42459_c0_g1_i1_3:134..213:+ |
| novel2 | 1874.9 | uggcaucugucgaggucaucua | TRINITY_DN44807_c0_g1_i1_3:303..372:+ |
| novel3 | 1794.4 | uuuugccugaaccgcccauacc | TRINITY_DN43786_c0_g2_i1_1:66..133:+ |
| novel4 | 1790.7 | ggaaugggcugauugacaaaa | TRINITY_DN33265_c0_g1_i1_2:547..624:- |
| novel5 | 1570.9 | uuuucccugaaccacccauucc | TRINITY_DN33188_c0_g1_i1_1:845..905:+ |
| novel6 | 1530 | uuaccgaucccuccaaagcc | TRINITY_DN52121_c1_g1_i3_3:1317..1383:- |
| novel7 | 1426.2 | aggggaugaccgaucugucugu | TRINITY_DN57876_c0_g1_i1_3:290..369:+ |
| novel8 | 1380 | uauauauucaggcugcuucuu | TRINITY_DN37640_c0_g1_i1_1:355..440:+ |
| novel9 | 1259.4 | uugccgaugccgcccauaccu | TRINITY_DN4591_c0_g1_i1_2:411..490:- |
| novel10 | 1259 | uuuuuccaauaccuccuauacc | TRINITY_DN3678_c0_g1_i1_1:302..368:- |
| novel11 | 1042.8 | ucuuaccugguccucccaugcc | TRINITY_DN32986_c0_g1_i1_2:27..103:+ |
| novel12 | 979.8 | uaugcagugcuguuuuucccauu | TRINITY_DN69460_c0_g1_i1_3:118..176:+ |
| novel13 | 906.1 | uuccucucgggguucuccaaau | TRINITY_DN55315_c0_g1_i2_3:892..971:- |
| novel14 | 891.7 | ucuuccccgauccucccaugcc | TRINITY_DN34805_c0_g1_i1_3:354..423:+ |
| novel15 | 884.5 | gcucaagaaagcuguggga | TRINITY_DN49780_c0_g1_i2_2:438..491:+ |
| novel16 | 840.8 | ucuugccugaaccgcccauacc | TRINITY_DN65523_c0_g1_i1_3:356..424:- |
| novel17 | 826.5 | uuuugccggucccuccaaugccu | TRINITY_DN63156_c0_g1_i1_2:54..122:+ |
| novel18 | 783.4 | uucccugauccucccaugccc | TRINITY_DN38574_c0_g1_i2_2:222..296:+ |
| novel19 | 654.7 | ucuuaccgaugccucccauucc | TRINITY_DN51819_c0_g1_i1_3:1128..1200:- |
| novel20 | 613.9 | uuugagugaauccagagucucu | TRINITY_DN34310_c0_g1_i1_3:2053..2135:- |
| novel21 | 612.7 | ucuuuccaauuccgcccauuc | TRINITY_DN66494_c0_g1_i1_3:721..792:- |
| novel22 | 587.4 | uugagccgcgccaauaucac | TRINITY_DN41620_c0_g4_i1_1:291..372:- |
| novel23 | 584.5 | uugagccgcgccaauaucac | TRINITY_DN64184_c0_g1_i1_2:-1..80:+ |
| novel24 | 540.2 | uggagaagcagggcacgugcg | TRINITY_DN30671_c0_g1_i1_3:381..438:- |
| novel25 | 476.3 | uccccucaaaggcuuccuaa | TRINITY_DN46366_c0_g1_i1_3:96..175:+ |
| novel26 | 449 | cuggcauucuguccaccucca | TRINITY_DN34748_c0_g1_i1_1:271..347:+ |
| novel27 | 421.8 | cuggcauucuguccaccucc | TRINITY_DN31814_c0_g1_i1_3:545..620:- |
| novel28 | 393.2 | auaaauuuagaucugucaggu | TRINITY_DN53352_c1_g4_i4_1:54..137:- |
| novel29 | 383.3 | ugccuggcucccuguaug | TRINITY_DN47404_c0_g1_i1_1:670..753:+ |
| novel30 | 359.1 | agugggaugcuaggaaagacu | TRINITY_DN45248_c0_g1_i1_1:162..243:+ |
| novel31 | 233.7 | uuucagaucuugaggacgcc | TRINITY_DN52471_c2_g1_i2_2:1666..1726:- |
| novel32 | 216.8 | uggucagaugagucauguuca | TRINITY_DN57680_c0_g1_i2_3:999..1050:+ |
| novel33 | 215.8 | agcugcaacaguugaacuugu | TRINITY_DN36068_c0_g1_i1_2:105..183:+ |
| novel34 | 180.6 | ucuuaccuauucccccuauucc | TRINITY_DN41938_c0_g1_i1_2:518..599:- |
| novel35 | 177.7 | aauaggaggucaggcaaagauu | TRINITY_DN33639_c0_g1_i1_1:104..185:+ |
| novel36 | 171.7 | cggaaccuuuguggggagaua | TRINITY_DN45447_c0_g1_i1_3:486..550:- |
| novel37 | 148.4 | agggcaauucuccuuuggcggg | TRINITY_DN2450_c0_g1_i1_1:156..234:+ |
| novel38 | 127.8 | cgauggaagauuuuugugcuc | TRINITY_DN53146_c1_g3_i2_2:1591..1678:- |
| novel39 | 106 | aaucucccucaaagguuucc | TRINITY_DN42508_c0_g1_i1_3:332..408:- |
| novel40 | 96.3 | uuuuuccuuccucuugacuac | TRINITY_DN52389_c0_g1_i2_3:154..238:- |
| novel41 | 85.6 | ucgcuggaagugcaucuucac | TRINITY_DN60014_c0_g1_i1_1:72..135:+ |
| novel42 | 84.8 | acaaauaguaaucauggcuucu | TRINITY_DN50054_c0_g1_i1_3:52..140:+ |
| novel43 | 83.8 | uugagcaugugggucugcagc | TRINITY_DN53312_c1_g2_i1_1:2696..2773:+ |
| novel44 | 78.4 | acauugugaucccuggauaga | TRINITY_DN52003_c0_g1_i2_1:1399..1489:+ |
| novel45 | 78.4 | acauugugaucccuggauaga | TRINITY_DN54814_c0_g1_i1_3:2909..2999:+ |
| novel46 | 75.6 | uugacagaagauagagggcac | TRINITY_DN30569_c0_g1_i1_1:357..439:+ |
| novel47 | 73.2 | uaagauuauguggagaagc | TRINITY_DN39431_c0_g1_i1_1:461..539:+ |
| novel48 | 73.2 | uaagauuauguggagaagc | TRINITY_DN24305_c0_g1_i1_2:417..495:- |
| novel49 | 62.8 | uuguagcuuggcuaaucucuua | TRINITY_DN34848_c0_g1_i1_3:7620..7698:+ |
| novel50 | 62.5 | ucaacauuucgaaucacacuc | TRINITY_DN53711_c0_g1_i2_3:1199..1245:- |
| novel51 | 58.6 | uucuggauuagauugugu | TRINITY_DN53570_c0_g1_i1_3:1830..1909:+ |
| novel52 | 46 | ggcgaaaugacugugugagaaa | TRINITY_DN38847_c0_g1_i2_3:264..348:- |
| novel53 | 45.7 | auuucccuucccaaauuuau | TRINITY_DN58646_c5_g1_i1_3:133..182:- |
| novel54 | 44.3 | uccagaaacaaaaguccaauu | TRINITY_DN43579_c0_g1_i1_1:1881..1923:- |
| novel55 | 38.9 | ccaaaggagaauugcccuu | TRINITY_DN2450_c0_g1_i1_1:155..234:- |
| novel56 | 29.8 | ugacuguuaucugugugagag | TRINITY_DN56163_c0_g1_i1_2:35..82:- |
| novel57 | 29.3 | cauuggugcauccuagggcu | TRINITY_DN63376_c0_g1_i1_3:27..107:- |
| novel58 | 28.1 | auucagaucgauacccaagag | TRINITY_DN57657_c5_g2_i1_3:449..535:+ |
| novel59 | 27.2 | uuucccucaggauagcua | TRINITY_DN58449_c0_g1_i2_3:1360..1441:+ |
| novel60 | 22.9 | uggcucaguucagcaggaacag | TRINITY_DN52517_c1_g1_i4_3:772..832:+ |
| novel61 | 18.4 | aaugaucucaacucuuguag | TRINITY_DN53588_c1_g1_i1_1:1590..1660:- |
| novel62 | 17 | aauggguggacgcggaagauu | TRINITY_DN58585_c1_g1_i1_3:58..118:+ |
| novel63 | 17 | aauggguggacgcggaagauu | TRINITY_DN53175_c1_g3_i1_2:50..110:+ |
| novel64 | 12.5 | ugacgaugguacuacuggaag | TRINITY_DN57324_c0_g1_i1_1:708..799:- |
| novel65 | 10.8 | aauuccuuggcugaaucuuuu | TRINITY_DN53220_c0_g1_i1_1:2139..2221:+ |
| novel66 | 7.8 | gcuuuccaugguaggaua | TRINITY_DN53853_c0_g1_i1_1:88..167:- |
| novel67 | 6.7 | cguguugugucaaauuaagcc | TRINITY_DN41037_c0_g1_i1_2:427..507:- |
| novel68 | 5.8 | ucuucguuuuaaguucucuua | TRINITY_DN43601_c0_g1_i1_1:156..219:+ |
| novel69 | 5.7 | agggcaauucuccuuugga | TRINITY_DN56502_c0_g1_i1_2:13..94:- |
| novel70 | 5.6 | ucuuaccugguccucccaug | TRINITY_DN48058_c0_g2_i1_3:535..609:- |
| novel71 | 5.6 | agccaaagauuagccagccccg | TRINITY_DN57721_c2_g2_i1_3:1209..1290:- |
| novel72 | 5.6 | uuuucccuguaccacccauu | TRINITY_DN56210_c0_g1_i1_2:1030..1089:+ |
| novel73 | 5.5 | uuccaucucuugcacacu | TRINITY_DN30726_c0_g1_i1_1:573..654:+ |
| novel74 | 5.5 | uucacaguggcuaaguucugc | TRINITY_DN71395_c0_g1_i1_3:58..121:+ |
| novel75 | 5.5 | uucacaguggcuaaguuccg | TRINITY_DN52517_c1_g1_i4_3:617..677:+ |
| novel76 | 5.4 | uugagccgugccaauaucacg | TRINITY_DN64385_c0_g1_i1_3:58..141:+ |
| novel77 | 5.4 | ucuuuccuuuaccaccgaua | TRINITY_DN22418_c0_g1_i1_2:895..964:- |
| novel78 | 5.3 | agcacuugacugguagcucgu | TRINITY_DN50890_c0_g2_i1_3:572..645:- |
| novel79 | 5.3 | ucuggaugcuggcucuguuccucc | TRINITY_DN21026_c0_g1_i1_2:29..119:- |
| novel80 | 5.3 | ucuugccauuccuccuaugcc | TRINITY_DN38574_c0_g1_i2_2:220..289:- |
| novel81 | 5.2 | ucagccuaggcugggaggugagca | TRINITY_DN1919_c0_g1_i2_1:721..777:+ |
| novel82 | 5.2 | ccucuuccucaaaggcucucaa | TRINITY_DN54337_c0_g1_i1_3:634..699:- |
| novel83 | 5.2 | uuuuuggagaguugaauggca | TRINITY_DN64473_c0_g1_i1_2:136..199:- |
| novel84 | 5.1 | uugccgaucagcccaugccu | TRINITY_DN46135_c1_g1_i1_2:352..427:- |
| novel85 | 5.1 | uugaacuuucuucagcuuacu | TRINITY_DN55987_c3_g1_i4_3:544..608:- |
| novel86 | 5.1 | ucacacaaccaagaagcacug | TRINITY_DN46352_c0_g1_i2_1:2019..2088:- |
| novel87 | 5 | ugaugauggaucauagagugc | TRINITY_DN57129_c0_g3_i1_3:573..644:+ |
| novel88 | 4.8 | ucuggaugcuggcucuguuccucc | TRINITY_DN21026_c0_g1_i1_2:95..162:- |
| novel89 | 4.6 | uugucgagucuuccgaaagga | TRINITY_DN58420_c1_g1_i13_3:2284..2365: |
| novel90 | 4.6 | uacauggauauucucugggga | TRINITY_DN24637_c0_g1_i1_1:275..338:+ |
| novel91 | 4.4 | auaaugaacucaugugaugug | TRINITY_DN58283_c2_g1_i1_3:524..594:+ |
| novel92 | 4.3 | uuuggauggucagaaaauau | TRINITY_DN57570_c0_g1_i1_2:1296..1367:+ |
| novel93 | 4.2 | auccaagacuauccuuaug | TRINITY_DN45962_c0_g1_i2_1:1996..2039:+ |
| novel94 | 3.9 | caauguuuugaaucacacuca | TRINITY_DN43790_c0_g1_i1_2:41..120:+ |
| novel95 | 3.8 | uaacugauuuaaugagccauuc | TRINITY_DN53786_c0_g1_i5_1:1128..1204:+ |
| novel96 | 3.5 | uuugaacccaugaucuaauu | TRINITY_DN46507_c1_g1_i1_3:428..481:+ |
| novel97 | 3 | ugauggaucauggaaugugaa | TRINITY_DN57129_c0_g3_i1_3:353..411:+ |
| novel98 | 2.9 | ugaugauggaucauagaguuu | TRINITY_DN52069_c0_g1_i2_1:1323..1381:- |
| novel99 | 2.9 | auguuugaauugacagggucc | TRINITY_DN19026_c0_g1_i1_3:604..683:+ |
| novel100 | 2.9 | auguuugaauugacagggucc | TRINITY_DN42177_c0_g1_i1_1:1732..1811:- |
| novel101 | 2.7 | acaaggaauugcagacgugau | TRINITY_DN51097_c0_g1_i1_2:1663..1713:+ |
| novel102 | 2.6 | aagaauuucgcccuggacccu | TRINITY_DN32104_c0_g1_i1_1:22..74:- |
| novel103 | 2.4 | ugagaagauggcuaucugcac | TRINITY_DN53761_c1_g2_i1_1:1266..1330:+ |
| novel104 | 2.3 | ucccaaggagacguggagaug | TRINITY_DN57302_c0_g1_i1_1:97..162:- |
| novel105 | 2.2 | ggauugugaugaauauaaagu | TRINITY_DN45064_c0_g1_i1_3:402..483:+ |
| novel106 | 2.2 | uccaccgucgguguugccacc | TRINITY_DN51329_c3_g1_i9_2:426..498:- |
| novel107 | 2.2 | uaauggauuuguuauuacuca | TRINITY_DN51938_c0_g2_i1_1:1082..1144:- |
| novel108 | 2.2 | ucuggcugaccaugaaccuca | TRINITY_DN41767_c0_g1_i2_2:516..590:+ |
| novel109 | 2.1 | uccaucugaaagacucgcg | TRINITY_DN58392_c3_g3_i2_3:4014..4075:+ |
| novel110 | 2.1 | aagggauagaguuucauuu | TRINITY_DN52121_c1_g1_i3_3:1459..1508:- |
| novel111 | 1.9 | cagugaggucacuuaggaca | TRINITY_DN53019_c0_g1_i3_3:333..387:- |
| novel112 | 1.9 | uugucaauaaauagaaaacaug | TRINITY_DN50208_c0_g2_i1_1:493..567:+ |
| novel113 | 1.8 | uccccucaaaggcuuccuaaa | TRINITY_DN35421_c0_g1_i1_3:85..168:+ |
| novel114 | 1.8 | uucuggauuugauuguguaua | TRINITY_DN11410_c0_g1_i1_3:496..551:+ |
| novel115 | 1.7 | uugccaguuccgcccauuccua | TRINITY_DN46135_c1_g1_i1_2:357..430:+ |
| novel116 | 1.7 | uaagauugauaucucuuggcc | TRINITY_DN45203_c0_g1_i1_3:41..125:+ |
| novel117 | 1.7 | ggguucaauagggcuauagu | TRINITY_DN61138_c0_g1_i1_3:418..475:+ |
| novel118 | 1.7 | uugucaaucagcccauuccu | TRINITY_DN33265_c0_g1_i1_2:550..625:+ |
| novel119 | 1.6 | ugaacuuucuucagcuuacu | TRINITY_DN49307_c0_g1_i1_1:467..531:- |
| novel120 | 1.5 | ucccggcaggcgugacuccuc | TRINITY_DN34357_c0_g1_i1_3:63..126:+ |
| novel121 | 1.5 | aucccucaaaggcuuccaa | TRINITY_DN45133_c0_g1_i1_2:325..410:- |
| novel122 | 1.4 | aucacauugccagggauu | TRINITY_DN52517_c1_g1_i4_3:438..488:+ |
| novel123 | 1.4 | auucugaugaagcuugacuag | TRINITY_DN48966_c0_g3_i1_3:23..102:+ |
| novel124 | 1.3 | uagucggccugcccugauggc | TRINITY_DN28811_c1_g1_i1_3:87..170:- |
| novel125 | 1.2 | agaagaucugacagaaauuuu | TRINITY_DN52655_c1_g5_i2_2:1078..1122:- |
| novel126 | 1.2 | uuggagccagaauagccgcagc | TRINITY_DN57168_c2_g1_i1_3:90..172:+ |
| novel127 | 1.2 | uuuucgaucauauuuugcaau | TRINITY_DN51583_c0_g1_i4_1:259..343:+ |
| novel128 | 1.1 | aguaugaucugaaacauugau | TRINITY_DN47201_c0_g2_i1_1:81..170:- |
| novel129 | 1.1 | cugaguacugcugaagcug | TRINITY_DN32173_c0_g1_i1_1:2338..2410:+ |
| novel130 | 1.1 | uuuuaccagaaccgcccauu | TRINITY_DN2296_c0_g1_i1_3:409..482:- |
| novel131 | 1.1 | ucuaaucuugcaaaacuugug | TRINITY_DN53330_c4_g2_i2_1:2524..2612:+ |
| novel132 | 0.9 | ucuauuuuuaaggaagaggug | TRINITY_DN53472_c3_g2_i1_1:2993..3077:+ |
| novel133 | 0.9 | ccgagcccaaguccccug | TRINITY_DN50408_c0_g1_i1_2:205..266:+ |
| novel134 | 0.8 | uggggaaugggaaaguca | TRINITY_DN58568_c1_g4_i1_3:281..359:+ |
| novel135 | 0.7 | ugaugauggaucauagagugc | TRINITY_DN57129_c0_g3_i1_3:623..685:+ |
| novel136 | 0.5 | uugagccgcgccaauaucgcc | TRINITY_DN67001_c0_g1_i1_3:1722..1773:- |
| novel137 | 0.4 | ugugucuaggauaucaaucuc | TRINITY_DN20428_c0_g1_i1_2:220..294:- |
| novel138 | 0.4 | aauacggcgcugcaugaagcu | TRINITY_DN56863_c0_g3_i3_3:1594..1642:- |
| novel139 | 0.4 | ugcugaugguaugacaacgcc | TRINITY_DN4041_c0_g1_i1_1:267..346:- |
| novel140 | 0.2 | uuuggaaucagggccucugug | TRINITY_DN67382_c0_g1_i1_3:235..308:- |
| novel141 | 0.2 | cgcacuguaccuccguca | TRINITY_DN4293_c0_g1_i1_3:232..280:- |
| novel142 | 0.1 | auaucugacuauuuauaug | TRINITY_DN52001_c0_g1_i1_2:3861..3922:- |
| novel143 | 0 | aaugucucaacgguacucuag | TRINITY_DN57749_c1_g1_i5_3:749..805:- |
| novel144 | 0 | uuugacccagagcuaugaaccggu | TRINITY_DN35145_c0_g1_i1_3:656..732:+ |
| novel145 | 0 | aaguaugggacugugacagcg | TRINITY_DN34983_c0_g1_i1_2:498..570:- |
